# The Onset of Interictal Spike-Related Ripples Facilitates Detection of the Epileptogenic Zone

**DOI:** 10.3389/fneur.2021.724417

**Published:** 2021-11-04

**Authors:** Yurika Numata-Uematsu, Mitsugu Uematsu, Rie Sakuraba, Masaki Iwasaki, Shinichiro Osawa, Kazutaka Jin, Nobukazu Nakasato, Shigeo Kure

**Affiliations:** ^1^Department of Pediatrics, Tohoku University School of Medicine, Sendai, Japan; ^2^Department of Epileptology, Tohoku University School of Medicine, Sendai, Japan; ^3^Department of Neurosurgery, Tohoku University School of Medicine, Sendai, Japan; ^4^Department of Neurosurgery, National Center Hospital of Neurology and Psychiatry, Tokyo, Japan

**Keywords:** surgery, epileptogenic zone, epilepsy, interictal, onset, spike-related ripples

## Abstract

**Objective:** Accurate estimation of the epileptogenic zone (EZ) is essential for favorable outcomes in epilepsy surgery. Conventional ictal electrocorticography (ECoG) onset is generally used to detect the EZ but is insufficient in achieving seizure-free outcomes. By contrast, high-frequency oscillations (HFOs) could be useful markers of the EZ. Hence, we aimed to detect the EZ using interictal spikes and investigated whether the onset area of interictal spike-related HFOs was within the EZ.

**Methods:** The EZ is considered to be included in the resection area among patients with seizure-free outcomes after surgery. Using a complex demodulation technique, we developed a method to determine the onset channels of interictal spike-related ripples (HFOs of 80–200 Hz) and investigated whether they are within the resection area.

**Results:** We retrospectively examined 12 serial patients who achieved seizure-free status after focal resection surgery. Using the method that we developed, we determined the onset channels of interictal spike-related ripples and found that for all 12 patients, they were among the resection channels. The onset frequencies of ripples were in the range of 80–150 Hz. However, the ictal onset channels (evaluated based on ictal ECoG patterns) and ripple onset channels coincided in only 3 of 12 patients.

**Conclusions:** Determining the onset area of interictal spike-related ripples could facilitate EZ estimation. This simple method that utilizes interictal ECoG may aid in preoperative evaluation and improve epilepsy surgery outcomes.

## Introduction

The epileptogenic zone (EZ) is characterized by excessive synchronization at seizure onset (1). It is generally located within the resection region following surgery and has seizure freedom (2, 3). The identification of the EZ, including the seizure onset zone (SOZ) (4), and the complete resection thereof are essential for improving epilepsy seizure outcomes. The area with the earliest ictal electrocorticography (ECoG) change is currently used to detect the SOZ (5). However, resection of the EZ identified based on ictal ECoG patterns leads to a seizure-free outcome in only about 60% of patients who undergo epilepsy surgery (6, 7). In an effort to improve seizure outcomes, many studies have reported that brain regions with high-frequency oscillations (HFOs; >80 Hz gamma oscillation activity) during ictus are likely within the EZ (8–10). Ictal direct current shifts preceding HFOs have recently been reported to be useful for EZ detection (11, 12).

As seizures are infrequent in some cases, recording ictal events can be difficult. Further, it remains unclear whether resection of the onset foci of interictal spikes would improve surgical outcomes (13). Some studies have identified the EZ using interictal HFOs (14–16), which are reportedly more specific than spikes for localizing the SOZ and show a good correlation with postsurgical outcomes in patients with epilepsy (5, 17, 18).

Nevertheless, a recent prospective multicenter trial reported that interictal HFOs do not predict postsurgical seizure outcomes (19). Gamma activity, including HFOs, is observed in a wide range of areas in the normal brain, including the eloquent area (20). Moreover, as interictal epileptic discharges propagate, it is necessary to consider which characteristic spikes or HFOs should be analyzed. Tamilia et al. recently reported that resecting areas with interictal HFO onset improved the prognosis of epileptic seizures (21). Although that study was concerned with interictal HFOs, physiological gamma activity was not ruled out. Wang et al. also reported that HFOs occurring simultaneously with epileptic spikes could be used to exclude physiological HFOs (22). However, the degree of spike-related high-frequency power augmentation failed to differentiate between SOZ and non-SOZ (23).

In the present study, we hypothesized that the onset area of ripples (HFOs of 80–200 Hz) related to interictal spikes propagating among electrodes could facilitate the detection of the EZ. To explore this hypothesis, we developed a method to determine the onset area of interictal spike-related ripples. Then, among patients who underwent surgical resection and achieved seizure-free outcomes, we retrospectively examined the relationship between ripple onset and the resection area, including the EZ.

## Materials and Methods

Between July 2011 and June 2014, we retrospectively identified patients with medically intractable epilepsy who underwent intracranial EEG monitoring and focal resective surgery within the Tohoku University Hospital Comprehensive Epilepsy Program who met the following inclusion criteria: resective surgery performed after intracranial EEG monitoring, with available postsurgical seizure outcome after 2 years, without seizure recurrence for 2 years after surgery, without previous brain surgery, and with a spike observed in the intracranial EEG. This study was approved by the Tohoku University Hospital Institutional Review Board (protocol numbers: A2017-028 and 2020-1-083). Informed consent was obtained from the patients or their guardians (for pediatric patients).

### Implantation of Intracranial Electrodes

Intracranial electrodes were implanted to monitor the epileptogenic area using scalp video-EEG recording, magnetic resonance imaging (MRI), and positron emission tomography (PET). Electrodes were implanted under general anesthesia guided by a neuro-navigation system (Brainlab AG, Feldkirchen, Germany). Subdural or strip electrodes, consisting of 2.3-mm-diameter platinum disk, exposed the area at an interelectrode distance of 10 mm (Ad-Tech Medical Instrument Corporation, Racine, WI, USA). Depth electrodes were inserted using a frameless stereotactic system (VarioGuide™; Brainlab AG). Depth electrodes were cylindrical platinum contacts 1.3 mm in length and 1.1 mm in diameter (Ad-Tech Medical Instrument Corporation). All electrode contacts were spaced at intervals of 5–15 mm.

### Extraoperative ECoG and EEG Recordings

Extraoperative video-ECoG recordings were obtained for 14 days using a Neurofax EEG-1200 instrument (Nihon-Koden Co., Tokyo, Japan). The sampling frequency was set at 1,000 Hz and the amplifier bandpass was at 0.08–300 Hz. For sleep staging, scalp electrodes were attached to 21 locations according to the International 10–20 system with electromyography (EMG)/electrooculography (EOG). For EMG, two electrodes were attached to the skin overlying the mentalis muscle. Two additional electrodes were attached to the left eye (upper left) and right eye (lower right) for electrooculography.

### Anatomical Locations of Electrodes and Surgical Resection

Three-dimensional computed tomography (3D-CT) and three-dimensional magnetization-prepared rapid-gradient echo (3D-MPRAGE) imaging were performed before electrode implantation and on day 7 after electrode implantation. These images were co-registered by linear affine transformation using Amira version 5 (Visualization Sciences Group, Inc., Burlington, MA, USA). Postoperative 3D-MPRAGE images were co-registered with the preoperative 3D-MPRAGE image and CT scan. The area of surgical resection and the relationship to the electrode location were visualized on the fusion image. Presurgical evaluation of the SOZ and resection area was performed by multiple epileptologists and epilepsy surgeons during a case review meeting.

### Markers of Interictal Spike Onset

ECoG recordings were visually reviewed to identify interictal epileptic spikes. For this analysis, ECoG segments were chosen during the non-REM sleep state without ictus to avoid contamination with motion artifacts. Periods of non-REM sleep were identified using scalp EEG, EMG, and EOG, as previously described (17).

**Figure 1:**
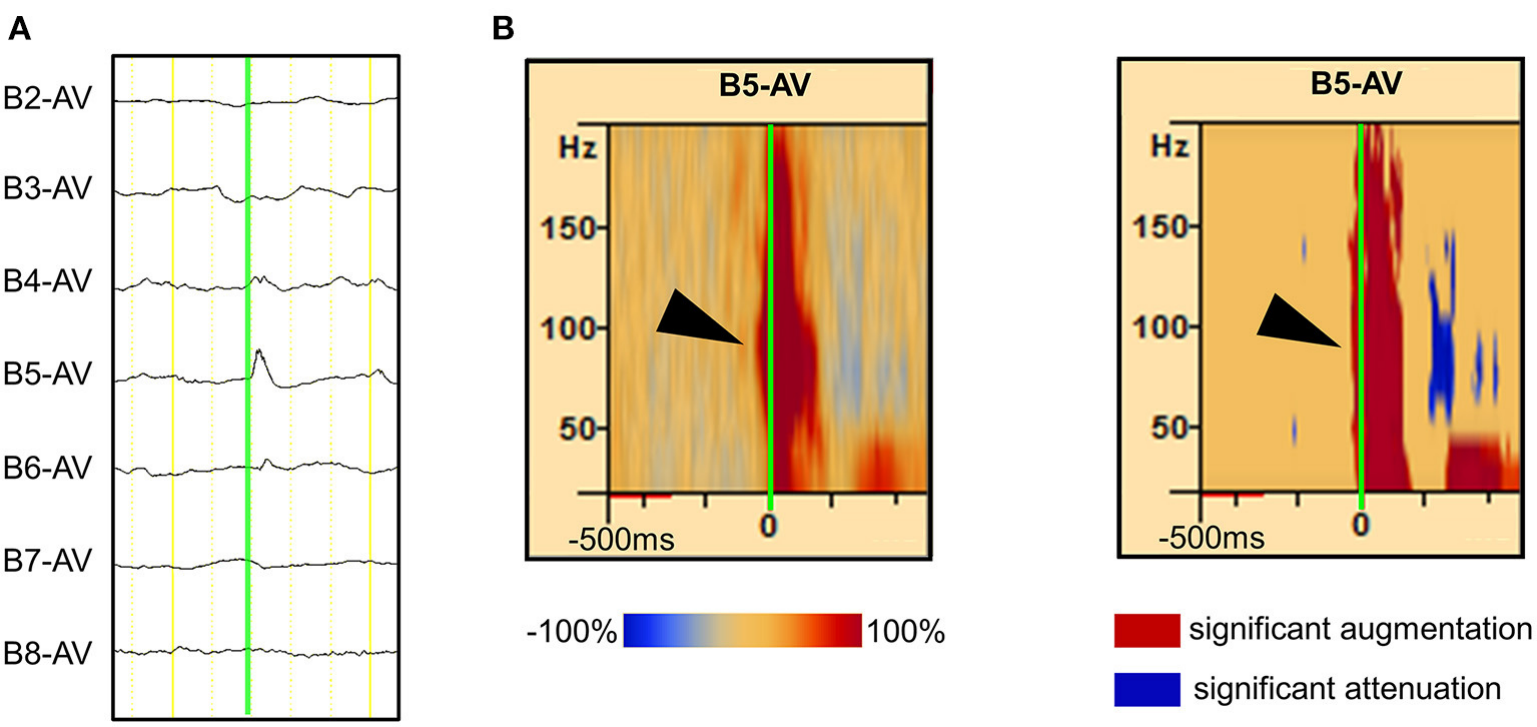
Detection of spike-related high-frequency oscillations (HFOs) using the BESA^®^ software. **(A)** The rising edge of the negative spike is marked (green line) in the channel of interest [channel (Ch) B5 is used as an example in this figure]. More than 30 spikes were marked in the same channel. (**B**, left) The average HFO augmentation associated with the spikes was analyzed using BESA^®^. Within the square, the “percentage augmentation” of the Ch B5 spike-related HFOs relative to the reference (300–500 ms prior to the spike, red bar) is shown, ranging from −100% (blue) to +100% (red). The earliest HFO augmentation was observed between 80 and 150 Hz (arrowhead). (**B**, right) The blue color indicates the significant attenuation and the red color indicates the significant augmentation of amplitude in a given time–frequency bin relative to the reference period. The analysis was done using studentized bootstrap statistics. The corrected α level was set to 0.05.

Interictal spikes were observed in the implanted channels. Three or fewer channels were selected in which frequent typical spikes with negative peaks were observed around the suspected SOZ. In each channel and spike set, 30 or more single spikes with almost the same morphology were visually marked at the point when the negative spike emerged from the baseline (Figure 1A). To ensure that the segments of the spectral analysis contained only one spike and its related spectral changes, only spikes with a minimum interspike interval of 500 ms were selected. For this reason, channels with continuous spiking or predominantly polyspike activity were excluded.

### Analysis of Interictal Spike-Related Gamma Activity

Visual detection of HFOs is challenging because they can have irregular amplitudes owing to the filtering of sharp epileptiform spikes (24). Generally, HFOs exhibiting at least four oscillations in the unfiltered signal, with an isolated peak in the time–frequency plane (blob), were considered as class 1 gamma activity events (Supplementary Figure 1A); and those with fewer than four oscillations or without blobs, were considered class 2 gamma activity events (Supplementary Figure 1B). In previous reports, class 2 gamma activity events were excluded because they may contain artifacts (25, 26). In the present study, class 1 gamma activity was defined as HFOs and further analyses were performed.

Each ECoG trial containing interictal spikes was transformed into the time–frequency domain using a complex demodulation technique applied using the BESA^®^ EEG V.5.1.8 software (BESA GmbH, Gräfelfing, Germany) (27, 28). The time–frequency transformation was obtained by multiplying the time domain signal with a complex exponential, followed by a low-pass filter. The low-pass filter used here was a finite impulse response filter with a Gaussian shape, rendering the complex demodulation effectively equivalent to a Gabor transform. For each trial, an ECoG signal was assigned an amplitude (a measure proportional to the square root of the power) as a function of time and frequency for each trial. Ripples were reported to have earlier ictal augmentation than those of <80 and >200 Hz (29). We did not analyze the HFOs above 200 Hz as it would be inaccurate, since the sampling frequency of the present study was set at 1,000 Hz. Time–frequency transformation was performed for frequencies between 20 and 200 Hz and latencies between −500 and 500 ms, relative to the onset of interictal spikes in steps of 10 Hz and 5 ms, respectively. For each time–frequency bin, we calculated the percent change in amplitude (averaged across trials) relative to the mean amplitude in a reference period of 300–500 ms prior to the onset of interictal spikes (Figure 1B, left). This change in amplitude has been termed temporal spectral evolution (TSE) (30). The TSE values were subjected to statistical tests using the BESA software (31–33). First, studentized bootstrap statistics were applied to obtain an uncorrected *p*-value for each time–frequency bin. This test compared the amplitude in each time–frequency bin with the average amplitude in the reference time period of the corresponding frequency. Second, correction for multiple testing was applied to these uncorrected *p*-values, to account for the fact that the TSE values in the neighboring time bins are partially dependent. For this purpose, a modified Bonferroni correction with a corrected α level of 0.05 was used (34). In all figures, blue indicates attenuation of amplitude, and red indicates augmentation of amplitude in the corresponding time–frequency bin relative to the reference period (Figure 1B, right).

We determined the proportion of sites showing significant amplitude modulation (at least 40 Hz in width and 20 ms in duration) in each patient (35). This correction has a very low probability of a Type I error when determining cortical activation or deactivation. We acknowledge that this analysis may underestimate gamma modulations when there is a restricted frequency band (<40 Hz in width) or signals of very short duration (<20 ms). Previous studies using scalp EEG recordings revealed augmentation of narrow-range gamma-band oscillations at ~40 Hz (36), whereas event-related gamma modulations observed in intracranial ECoG studies commonly involved frequency bands up to 20 Hz in width (37, 38). In the present study using ECoG, we defined at least 40 Hz in width and 20 ms in duration as significant gamma oscillation activity, which is more reliable for analyzing EZ than 20 Hz in width and 20 ms in duration.

In this study, the selection of electrode channels, marking of spikes, and analysis of ripple onset channels were performed by researchers who have not participated in the presurgical evaluation.

## Results

### Patient Characteristics

We studied consecutive records of 12 of the 26 patients who satisfied the inclusion criteria and underwent intracranial EEG monitoring during this period. The remaining 14 patients were excluded due to residual seizures (12 patients), few spikes during ECoG monitoring (1 patient), and no surgical resection (1 patient). Patients who did not become seizure-free after epilepsy surgery were excluded from this study because it was presumed that the EZ was not fully included within the implanted intracranial electrodes. The patients' clinical characteristics, including age at surgery, pathological findings, location and number of implanted electrodes, number of seizure onset electrodes, number of marked spikes, number of ripple-onset electrodes, and number of resection electrodes are summarized in Table 1. Surgical resection was performed on patients aged 14–39 years (median age, 31 years). In 10 patients, an abnormal brain lesion was detected on MRI: three patients had focal cortical dysplasia (FCD), two had hippocampal atrophy, one had FCD and hippocampal atrophy, one had tuberous sclerosis, one had trauma and hippocampal atrophy, one had hippocampal atrophy and a parahippocampal cystic lesion, and one had an amygdala enlargement. The two remaining patients had no abnormal MRI findings, with or without focal hypometabolism on PET. The pathological findings of the resected specimens from the 12 patients were FCD (*n* = 5), FCD and hippocampal sclerosis (*n* = 3), cortical tuber (*n* = 1), hippocampal sclerosis (*n* = 1), oligodendroglioma (*n* = 1), and inflammatory changes (*n* = 1). Nine of the 12 patients had undergone temporal lobectomy, and eight had also undergone hippocampal resection (Supplementary Table 1).

**Table 1:** Patients' characteristics and implanted electrodes data.

**Patient**	**Age at surgery (years)**	**Pathology**	**Electrode location**	**Number of electrodes**						**Ripple frequency at onset (Hz)**
				**Implant**	Seizure onset ^*^1	Marked spikes ^*^2	Ripple onset ^*^2	**Coincidence of seizure and ripple onset**	**Resection**	
1	14	Cortical tuber	Rt P, T, O	134	5	2	2	1	18	80–130
2	15	FCD	Lt P, T, O, and hippocampus	104	4	3	4	0	31	80–150
3	18	FCD	Lt P, T, O, and hippocampus	54	3	2	3	0	29	80–130
4	19	FCD	Rt F	52	3	1	1	0	8	80–150
5	27	Hippocampal sclerosis	Lt F, P, T, and hippocampus	78	7	3	4	0	46	80–150
6	28	FCD hippocampal sclerosis	Lt T and hippocampus	68	2	1	1	0	34	100–150
7	35	FCD	Rt F, bilateral T, and hippocampus	82	2	2	2	0	20	80–140
8	35	Inflammatory change	Rt T and hippocampus	28	7	1	1	1	25	80–150
9	35	Oligodendroglioma	Rt P, T, O	104	1	1	1	0	38	80–120
10	36	FCD hippocampal sclerosis	Lt T, and hippocampus	48	3	1	1	1	29	80–130
11	36	FCD hippocampal sclerosis	Rt F, bilateral T, and hippocampus	38	9	2	5	0	30	80–150
12	39	FCD	Rt F, T	36	3	1	1	1	17	80–150

*F, frontal; FCD, focal cortical dysplasia; Lt, left; O, occipital; P, parietal; Rt, right; T, temporal*.

*1 *Presurgical evaluation by multiple epileptologists and epilepsy surgeons*.

*2 *Selection of electrode channels, marking of spikes and analysis of ripple onset channels by researchers who did not participate in the presurgical evaluation*.

### Measurement of the Gamma-Onset Channel Relative to Interictal Spikes

**Figure 2:**
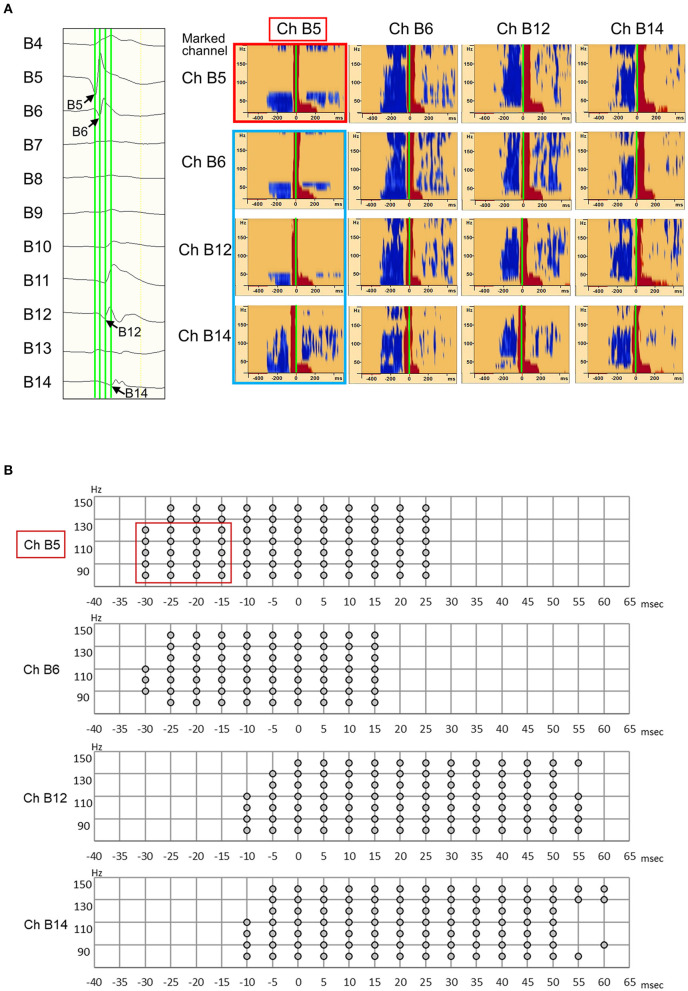
Illustration showing high-frequency oscillation (HFO) onset in Ch B5 for all marked channels (B5, B6, B12, and B14). (**A**, left) Interictal electrocorticography (ECoG) traces are shown, along with interictal spike propagation. In the enlarged ECoG trace, marks (green line) are attached to the rising edge of the Ch B5, Ch B6, Ch B12, and Ch B14 spikes. (**A**, right) The Ch B5, Ch B6, Ch B12, and Ch B14 spike-related HFOs are shown at the top, second from top, third from top, and at the bottom, respectively. The blue color indicates significant HFO attenuation and the red color indicates significant HFO augmentation in a given time–frequency bin relative to the reference period. At the top, the HFO onset of Ch B5 spike-related HFOs were observed as Ch B5 (red frame). In the other three channels, spike-related HFOs also have their onset in Ch B5 (blue frame). Thus, HFO onset can be accurately identified as Ch B5 by marking any channel (Ch B5, Ch B6, Ch B12, and Ch B14) over which the spikes spread. **(B)** Spike-related ripple onset in Ch B5. In our novel method, we defined significant HFO amplitude modulation as at least 40 Hz in width and 20 ms in duration. Spike-related ripple onset in Ch B5, with a latency of −30 ms, from 80 to 130 Hz (red frame). The spike-related ripples of Ch B6, Ch B12, and Ch B14 had a slightly longer latency than those of Ch B5.

Interictal epileptic spikes and gamma activity propagate from the epileptic focus when epileptic excitability occurs in the EZ. First, we investigated whether the identification of onset electrodes of spike-related gamma activity using our method could be altered by the selected electrode in a spike that spreads from one focus to multiple electrodes. We placed “tags” on the rising edge of each spike propagating across multiple electrodes. Time–frequency analysis conducted using the BESA software showed that the same channel always had the shortest gamma latency (Figure 2A). This result showed that, in the case of spikes emanating from one epileptic focus, the gamma-onset channel can be accurately identified by marking any channel over which the spikes spread. According to the results of the visual analysis, the earliest HFO augmentation was mostly in the range of 80–150 Hz (Figures 1B, 2B).

### Relationship Between Resection of Ripple Onset Channels and Epilepsy Surgery Outcomes

We investigated all implanted channels of the spike-related ripples and measured the onset of ripples in 12 patients with seizure-free outcomes after focal resection.

**Figure 3:**
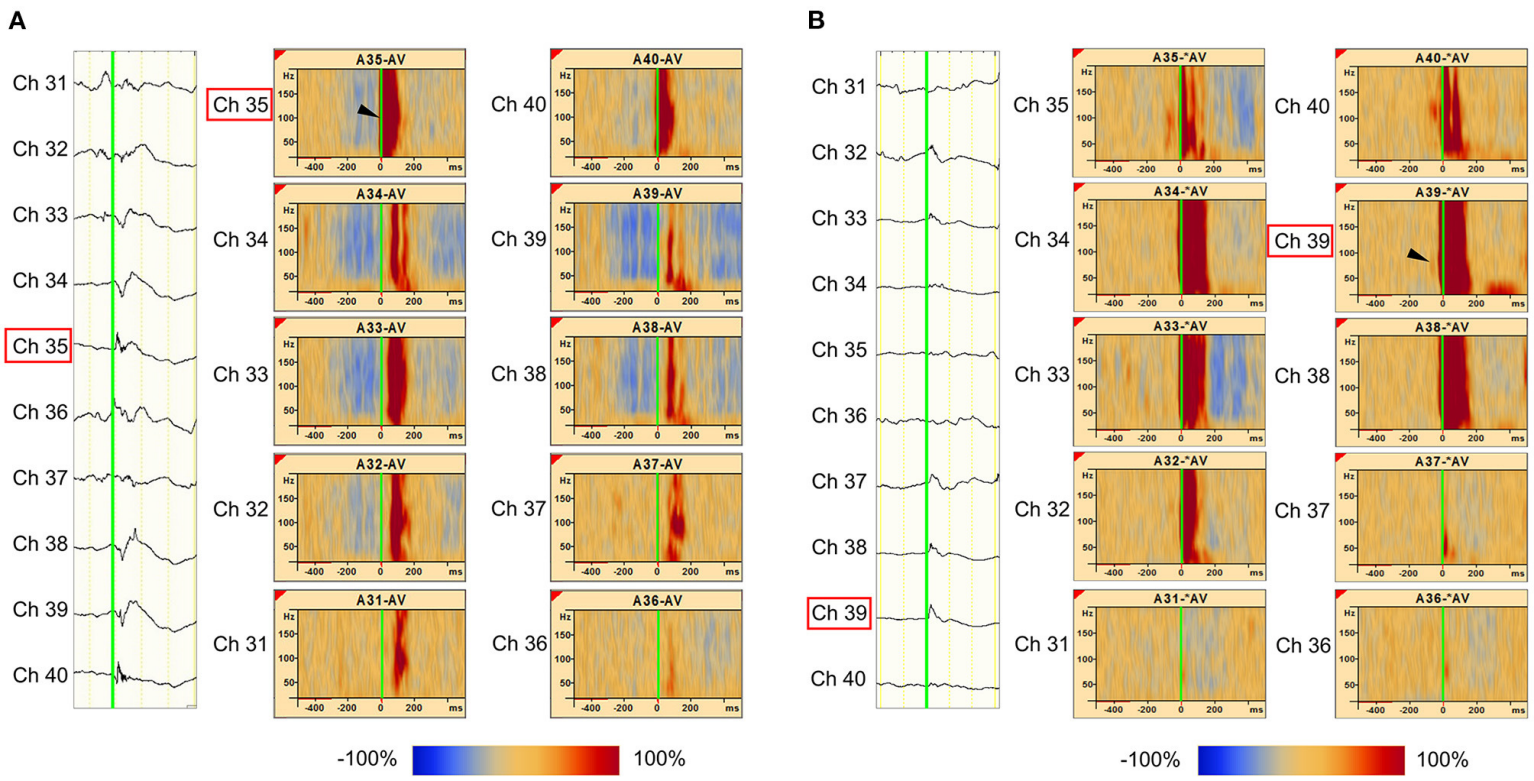
Marked interictal spikes and spike-related ripples in Patient 1. Green lines on the rising edge of channel A35 (Ch 35) [negative spike (**A**, left)] and channel A39 (Ch 39) [negative spikes (**B**, left)] are shown. The time–frequency plots in the square show the augmentation of ripples related to Ch 35 spikes (**A**, right) and Ch 39 spikes (**B**, right). Augmentation of ripples ranges from −100% (blue) to 100% (red) relative to the reference period (−500 to −300 ms). In the middle column of Ch 35 spike-related ripples **(A)**, the red bands from Ch 35 to Ch 31 show a progressively longer latency, which indicates propagation of ripples across these channels. The shortest latency of the Ch 35 spikes-related ripple augmentation channel (ripple onset) was visually estimated as Ch 35 (**A**, arrowhead), and the Ch 39 spikes-related ripple augmentation channel (ripple onset) was estimated as Ch 39 (**B**, arrowhead).

**Figure 4:**
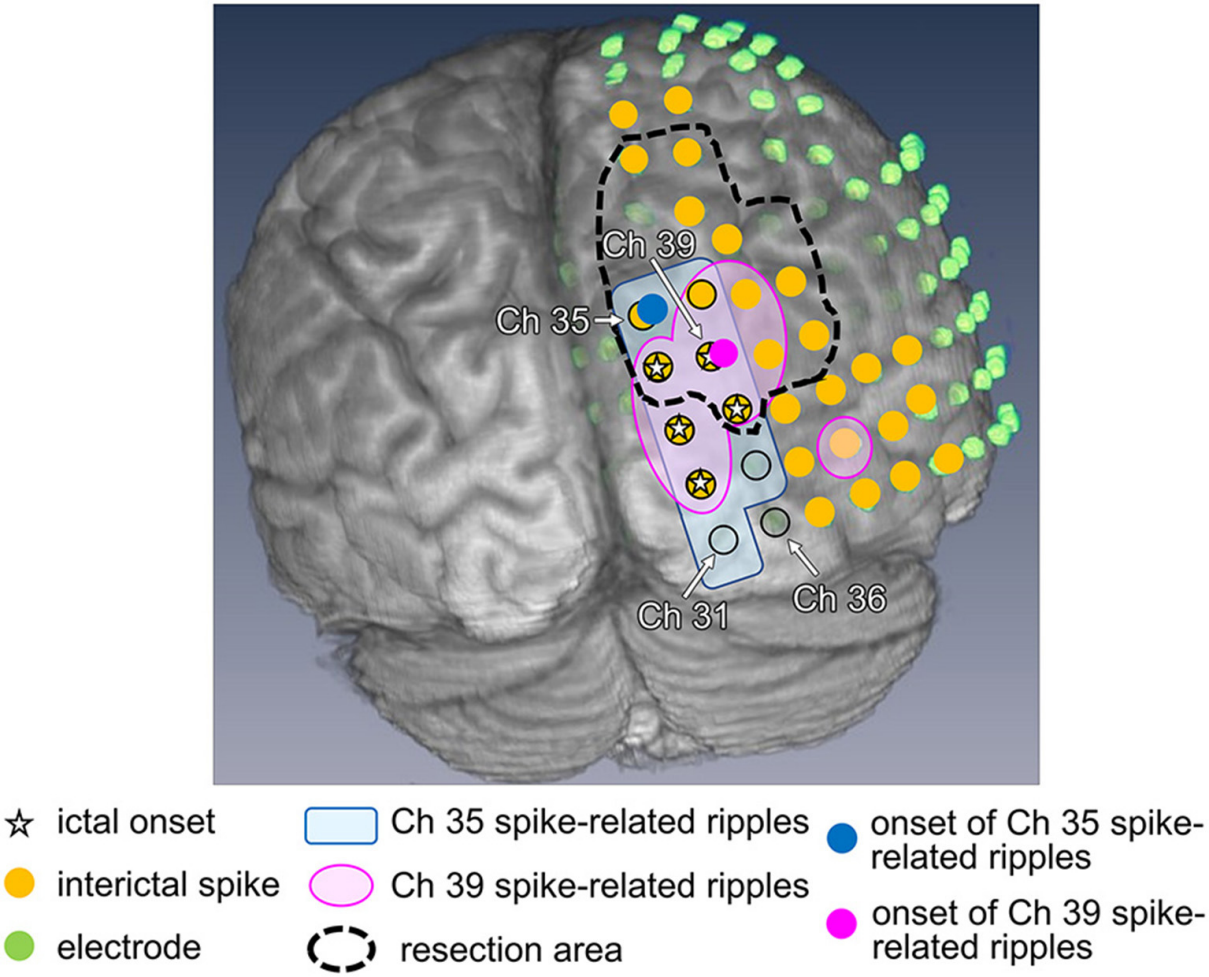
Ripple onset in Ch 35 and Ch 39, and other findings in Patient 1. Spike-related ripples of Ch 35 and Ch 39 (light pink and light blue, respectively), ripple onset in Ch 35 and Ch 39 (filled pink circles and filled blue circles, respectively), ictal onset (star), interictal spike (filled yellow circle), placed electrode (filled green circle), and the resection margin (black dotted line) are shown. The spike-related ripples of each channel propagated around the ictal onset area and had a narrower distribution than the spikes. Ch 35 is not estimated as the ictal onset, but rather as the ripple onset. Ch 39 was estimated as both ictal and ripple onset.

As an example, Patient 1 was a 14-year-old girl diagnosed with tuberous sclerosis complexes. She had a right occipital tuber and developed refractory epilepsy. During the electrocorticogram study, 134 electrodes were implanted from the right parietotemporal to the occipital area. Resection margins were independently determined based on ictal ECoG and were evaluated by several epileptologists and epilepsy surgeons. The spikes in channel 35 (Ch 35) (Figure 3A) showed different spread patterns from those in channel 39 (Ch 39) (Figure 3B). We marked the emergence of each spike from the baseline (Figures 3A,B, green line). Time–frequency plots derived from Ch 35 and Ch 39 in Patient 1 are shown in Figure 3. The onset channels of ripples determined by marking the spikes of Ch 35 and Ch 39, were denoted as Ch 35 (Figure 3A) and Ch 39 (Figure 3B), respectively. The spike-related HFOs appeared slightly before the marked spikes (Figure 3), and the onset latencies preceding the marked spikes were −10 and −15 ms in Ch 35 and Ch 39, respectively (Supplementary Table 1). In addition, gamma activity spread to the surrounding channels, with different onset latencies for each channel (Figures 3, 4). This patient appeared to have two different spike-onset channels with distinct HFO spread patterns.

**Figure 5:**
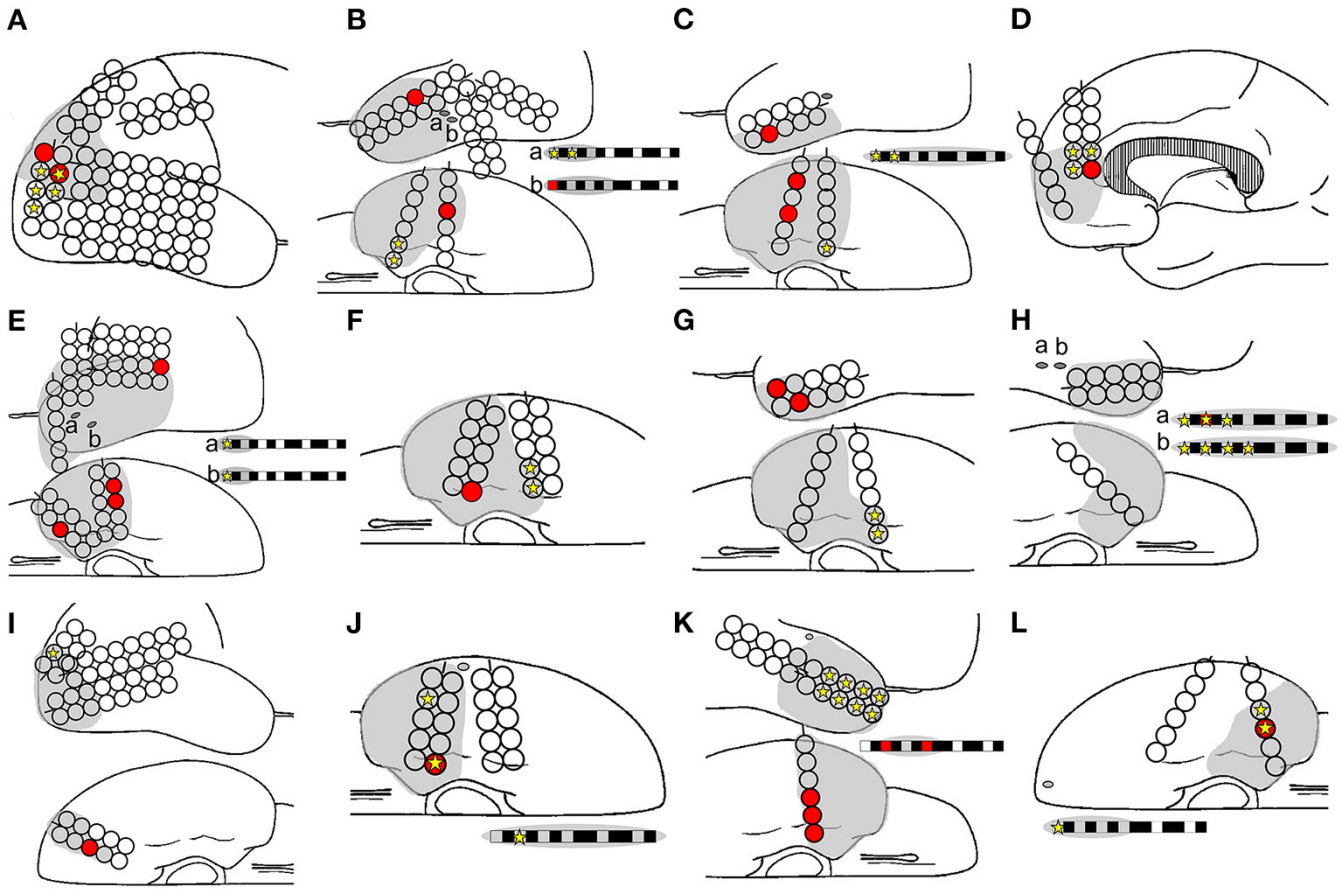
Relationships among ripple onset, ictal onset, and the resection area. The ripple onset electrode (filled red circle and box), ictal onset electrode (filled yellow star), and resection area (gray area) are shown. Letters **(A–L)** in the schema represent Patients 1–12. The ripple onset channel was within the resection area in all the patients. The ripples and ictal channels were identical for only three patients: Patients 8 **(H)**, 10 **(J)**, and 12 **(L)**.

In total, 1,068 analyzed ripples with interictal spikes were identified in 12 patients. The ripple onset channels were detected using one to five electrodes (Table 1, Supplementary Table 1). The ripples modulated by interictal spikes were widely spread over multiple channels but tended to be narrower than those modulated by spikes. Ripple onset frequencies were observed in the range of 80–150 Hz, especially in the range of 100–120 Hz, in all cases (Table 1, Supplementary Table 1). The onset latencies of ripples from 150 to 200 Hz were slightly longer than those of ripples from 80 to 150 Hz. One patient (Patient 12) exhibited a ripple onset from 150 to 200 Hz that was slightly shorter than that from 80 to 150 Hz (Supplementary Table 2). The average ripple onset latency from the spike of the marked channel was −22.8 ms (−125 to 15 ms) (Supplementary Table 1). The ripple and seizure onset channels also coincided in three patients (Patients 8, 10, and 12), and the ripple onset channels were near the seizure onset channels in the other nine patients. In one patient (Patient 1), the rate of coincided distribution of the resection area and the ripple onset channels was 50% (Figure 5, Supplementary Tables 1, 3, Supplementary Figure 2). Statistical analyses of onset latency revealed that the onset channels of spike-related ripples were within the surgical resection area in all patients (Figure 5, Supplementary Figure 2).

### Analyses of All Marked Spikes

We first marked 1,161 spikes in 12 patients, categorizing 1,068 spikes as class 1 (ripples) and 93 spikes as class 2 (Supplementary Table 1). On a pilot basis, we examined the onset of interictal spike-related ripples and class 2 gamma activity in all patients. The latency analysis showed almost the same results as those based on ripples. All gamma-onset channels (Supplementary Table 1) were within the resection area in all 12 patients (Supplementary Figure 2).

## Discussion

This study showed that all onset channels of interictal spike-related ripples were among the resection channels in 12 patients who were seizure-free after resective surgery. The ictal onset channels evaluated by ictal ECoG and the ripple onset channels were concordant in only three of the 12 patients. Therefore, our novel method based on interictal ECoG may facilitate the detection of the EZ, thus improving preoperative evaluations and epilepsy surgery outcomes.

### Epileptogenic Zone Estimated by Interictal Spikes

The EZ is a theoretical concept involving the area of the cortex, which is indispensable for the generation of epileptic seizures. In actual clinical practice, it is defined as the minimal cortical area that must be resected to produce seizure freedom (39). To evaluate the EZ preoperatively, EEG waveform patterns during seizures have been compared with ictal onset patterns such as low-voltage fast-activity (6, 40), which has a seizure elimination rate of about 60% (7). Recently, the usefulness of high-gamma activity (9, 41) and ictal direct current (11, 12) during seizures using time–frequency analysis has been reported. Changes in frequency during seizures have been more recently reported to be associated with EZ, based on analysis using stereoelectroencephalography (sEEG) (2, 3). Qi et al. evaluated EZ by HFO analysis during seizure using sEEG and reported that 16 of 19 patients (84%) they evaluated were seizure-free after surgery (42).

Our results indicate that the regions of interictal ripple onset are correlated with the EZ. ECoG abnormalities are often widely distributed during interictal spikes. Several clinical studies have suggested that interictal EEG findings are important in the context of epilepsy surgery. Concerning detection of the epileptic focus based on interictal spikes, previous studies measuring spike latency during interictal seizures reported a 53–77% probability of the spike-onset zone being within the EZ (43, 44). Hufnagel et al. found that short onset latency was more useful than spike frequency in estimating seizure focus (45). Further, Khoo et al. reported that spike-onset zone analysis using EEG/functional MRI was able to predict seizure focus with more than 90% accuracy (46). Using HFOs, Tamilia et al. also reported that resection of areas including the interictal HFO onset improved the prognosis of epileptic seizures (21). However, only 20% of these HFOs were related to spikes during interictal recording. Wang et al. reported that neocortical ripples superimposed on epileptiform discharges, such as paroxysmal fast, spike, or sharp waves, are specific markers of the SOZ, although many neocortical ripples are insufficiently localized (22). Therefore, we believe that our method of investigating the onset area of interictal spike-related ripples is reasonable for analyzing pathological HFOs generated within the EZ.

### Discrepancies Between Ictal Onset and Spike-Related Ripple Onset

Our results showed that the ictal and ripple onset channels completely coincided in only three of the 12 patients (rate of coincided distribution of ripple onset channels and ictal onset channels: 100%). Moreover, no common features, whether pathological or spatial, were observed among these three patients. Their pathological findings showed inflammatory changes, FCD and hippocampal sclerosis, and FCD alone. Moreover, the resected areas for these patients were the right temporal lobe and hippocampus, left temporal lobe and hippocampus, and right frontal and temporal lobes, respectively. These results did not exhibit a trend that is consistent with the other patients who did not have coinciding ictal onset channels (rate of coincided distribution of ripple onset channels and ictal onset channels: 0%). Maharathi et al. have previously described the spike origin, and have shown that EZ appears to be distinct in most cases (13). Nevertheless, we cannot definitively determine the cause of discrepancies between ictal onset and interictal spike-related ripple onset channels. In Patient 1, all interictal spike-related ripple onset channels were resected, and the patient became seizure-free; however, two of the five ictal onset electrodes assessed through ictal ECoG were not included in the resection area. Ultimately, we believe that our method of estimating the EZ from the onset of ripples using interictal ECoG could complement estimations of the EZ based on ictal ECoG.

### Simple Method for Detecting Pathological Gamma Activity

Since HFOs are usually observed as physiological brain activity, especially in primary sensory areas, it is necessary to determine whether they are related to epileptic activity in order to identify the focus of epilepsy. We believe that physiological HFOs can be excluded using our method because the HFOs that they analyze are associated with spikes (22). Moreover, the reference range was set from −500 to −300 ms before the onset of spikes during sleep.

Our data showed that the frequency range of spike-related ripple onset was 80–150 Hz. The onset latencies of ripples from 150 to 200 Hz were slightly longer than those from 80 to 150 Hz among almost all the analyzed channels. Moreover, in two patients (Patients 1 and 6), ripple onset channels of 150–200 Hz were not included in the resection area. Although only one patient (Patient 12) showed that the ripple onset from 150 to 200 Hz was slightly (10 ms) shorter than that from 80 to 150 Hz, the ripple onset channels of both frequency bands were of the same channel. These results suggest that ripples of 80–150 Hz are more useful for the EZ estimation than that of 150–200 Hz. Similarly, Nariai et al. reported that ripples in the range of 80–200 Hz had shorter ictal augmentation latency than those at <80 or >200 Hz (29). High-frequency activity in the range of 80–150 Hz has been related to epileptic activity (47, 48) and pathological multiunit burst firing during neocortical ictal activity (49, 50). These reports support our results showing that ripple onset frequencies range from 80 to 150 Hz.

Approximately 92% of the spikes (1,068 of 1,161) in this study were categorized as class 1 (ripple). This rate is probably higher than previously reported (56%) (25) because the spikes analyzed in the present study were more frequent and pathological. Additional studies of both class 1 and 2 gamma activities showed almost the same results as those with class 1 gamma activity only. All of the onset channels of interictal spike-related gamma activity were among the resection channels. Burnos et al. reported that separating real HFOs from “false oscillations” produced by the filtering effect of sharp spikes did not improve the delineation of the EZ (51). Although analyses through automatic detection of HFOs using software programs have also been conducted (52), in clinical applications, checking four or more oscillations or blobs for gamma activity associated with every spike on every electrode requires a lot of time. Our pilot study also supports the notion that there may be no need to distinguish false HFOs. This finding therefore demonstrates the simplicity of clinical HFO analysis.

### Limitations

This study had several limitations. First, it was retrospective and had a small sample size. Second, the propagation of gamma activity can only be observed in the part of the brain where the electrodes are located. Therefore, if there is no EZ in the area where the electrodes are located, our method cannot be used to estimate the EZ. Third, channels with spike intervals of <500 ms could not be analyzed because the reference period was set from −500 to −300 ms. Therefore, channels with very frequent spike activity could not be marked.

### Conclusion

We demonstrated that short-term interictal epileptic discharge recordings may facilitate identification of the EZ, even when no seizures are recorded. Even experienced epilepsy specialists can sometimes find it difficult to determine the resection area. Many parameters, such as seizure semiology and brain MRI and PET findings, are used to evaluate the EZ and surgical margins, whereas interictal ECoG spike findings are underutilized. Based on our findings, the onset area of interictal spike-related ripples could be useful for estimating the EZ. Since long-term intracranial electrode placement can be difficult in pediatric patients, we believe this method is especially useful for children.

## Data Availability Statement

The raw data supporting the conclusions of this article will be made available by the authors, without undue reservation.

## Ethics Statement

This study was approved by the Tohoku University Hospital Institutional Review Board (protocol numbers: A2017-028 and 2020-1-083). Written informed consent to participate in this study was provided by the participants' legal guardian/next of kin.

## Author Contributions

MU and YN-U designed the study, performed the experiments, analyzed the data, and wrote the manuscript. RS, MI, SO, and KJ performed the experiments, analyzed the data, and wrote the manuscript. NN and SK analyzed the data and wrote the manuscript. All authors contributed to the article and approved the submitted version.

## Funding

This study was partially supported by a Japan epilepsy research foundation grant (2014). The funders had no involvement in the study design, the collection, analysis, and interpretation of the data, the preparation of the report, or the decision to submit the manuscript for publication.

## Conflict of Interest

The authors declare that the research was conducted in the absence of any commercial or financial relationships that could be construed as a potential conflict of interest.

## Publisher's Note

All claims expressed in this article are solely those of the authors and do not necessarily represent those of their affiliated organizations, or those of the publisher, the editors and the reviewers. Any product that may be evaluated in this article, or claim that may be made by its manufacturer, is not guaranteed or endorsed by the publisher.
