# Supplementary material for: The Onset of Interictal Spike-Related Ripples Facilitates Detection of the Epileptogenic Zone
Source: Front Neurol. 2021 Nov 4;12:724417. doi: 10.3389/fneur.2021.724417 (PMC8599368; doi:10.3389/fneur.2021.724417)
Supplement: Supplementary file 1 [file Table_1.DOCX]

| Patient | MRI | PET | Resection area | Analyzed number of spikes | | | Marked channels | Ripple onset channels | Ripple onset band (Hz) | Ripple onset latency preceding a marked spike (ms) | Gamma onset channels  (all spikes) |
| --- | --- | --- | --- | --- | --- | --- | --- | --- | --- | --- | --- |
|  |  |  |  | class 1 (ripples) | class 2 | total |  |  |  |  |  |
| 1 | Rt occipital tuber with multiple calcified tubers | Rt occipital hypometabolism | Rt P, O | 53 | 0 | 53 | A35 | A35 | 80–130 | -10 | A35 |
|  |  |  |  | 64 | 7 | 71 | A39 | A39 | 80–130 | -15 | A39 |
| 2 | Lt temporal FCD  Lt hippocampal atrophy | Lt temporal hypometabolism | Lt T and  hippocampus | 56 | 7 | 63 | A13 | A13 | 80–150 | -15 | A7 |
|  |  |  |  | 48 | 7 | 55 | A34 | A34 | 80–140 | 0 | A34 |
|  |  |  |  | 69 | 5 | 74 | B5 | B5 | 80–130 | -25 | B5 |
|  |  |  |  |  |  |  |  | B6 | 80–140 |  |  |
| 3 | Lt hippocampal atrophy | Lt temporal hypometabolism | Lt T and  hippocampus | 53 | 1 | 54 | A9 | A33 | 80–130 | -10 | A7, A36 |
|  |  |  |  | 52 | 1 | 53 | A35 | A35 | 80–130 | -25 | A7, A35 |
| 4 | Rt medial frontal FCD | Rt medial frontal and basal temporal hypometabolism | Rt F | 45 | 10 | 55 | A7 | A6 | 80–150 | 15 | A6 |
| 5 | Lt hippocampal atrophy  Lt temporo-parietal traumatic scar | Lt centro-parieto-temporal  hypometabolism | Lt T and  hippocampus | 54 | 6 | 60 | A3 | A3 | 80–130 | -20 | A3 |
|  |  |  |  | 50 | 11 | 61 | A18 | A46 | 80–120 | 0 | A19 |
|  |  |  |  | 61 | 6 | 67 | B11 | A18 | 80–150 | -25 | A18  A19 |
| 6 | Lt hippocampal atrophy  Lt parahippocampal cystic lesion | Lt antero-medial temporal hypometabolism | Lt T and  hippocampus | 30 | 2 | 32 | A6 | A6 | 100–150 | -10 | A6 |
| 7 | normal | normal | Lt T and  hippocampus | 66 | 0 | 66 | B4 | B19 | 80–140 | -65 | B19 |
|  |  |  |  | 58 | 0 | 58 | B30 | B13 | 80–140 | -115 | B13 |
| 8 | Rt amygdala enlargement | Rt amygdala and hippocampus hypometabolism | Rt T and  hippocampus | 49 | 4 | 53 | A2 | A2 | 80–150 | 5 | A2, A3 |
| 9 | Rt occipital FCD | Rt temporo-occipital hypometabolism | Rt P, O | 68 | 0 | 68 | A38 | A33 | 80–120 | -20 | A33 |
| 10 | Lt mild hippocampal atrophy | Lt antero-medial temporal hypometabolism | Lt T and  hippocampus | 27 | 5 | 32 | A6 | A6 | 80–130 | -5 | A6 |
| 11 | normal | Rt antero-medial temporal hypometabolism | Rt T and  hippocampus | 74 | 5 | 79 | A1 | A40 | 80–130 | -125 | A31  A33  A40 |
|  |  |  |  | 56 | 1 | 57 | A38 | A31 | 80–130 | 0 | A31  A32  A33 |
|  |  |  |  |  |  |  |  | A32 | 80–130 |  |  |
|  |  |  |  |  |  |  |  | A33 | 80–130 |  |  |
|  |  |  |  |  |  |  |  | A38 | 80–150 |  |  |
| 12 | Rt occipital FCD | Rt antero-medial temporal hypometabolism | Rt T | 35 | 15 | 50 | A23 | A23 | 80–150 | 10 | A23 |

| 1068 | 93 | 1161 |
| --- | --- | --- |

F, frontal; FCD, focal cortical dysplasia; Lt, left; O, occipital; P, parietal; Rt, right, T, temporal.

Table S1. Patient's images and analyzed channels data
