# Supplementary material for: The Onset of Interictal Spike-Related Ripples Facilitates Detection of the Epileptogenic Zone
Source: Front Neurol. 2021 Nov 4;12:724417. doi: 10.3389/fneur.2021.724417 (PMC8599368; doi:10.3389/fneur.2021.724417)
Supplement: Supplementary file 2 [file Table_2.DOCX]

| Patient | Marked channels | Ripple onset channels  150–200Hz | Ripple onset channels  80–150Hz | Ripple onset band (Hz) | Ripple onset latency preceding a marked spike (ms) | Ripple onset latency preceding a marked spike (ms) | Is the Ripple onset channels  containing in resection electrodes? | |
| --- | --- | --- | --- | --- | --- | --- | --- | --- |
|  |  |  |  |  | 150–200Hz | 80–150Hz | 150–200Hz | 80–150Hz |
| 1 | A35 | A35 |  | 150–200 | -5 |  | Yes | Yes |
|  |  |  | A35 | 80–130 |  | -10 | Yes | Yes |
|  | A39 | A33 |  | 150–200 | -5 |  | **No** | Yes |
|  |  | A34 |  | 150–200 | -5 |  | Yes | Yes |
|  |  |  | A39 | 80–130 |  | -15 | Yes | Yes |
| 2 | A13 | A13 |  | 150–200 | -5 |  | Yes | Yes |
|  |  |  | A13 | 80–150 |  | -15 | Yes | Yes |
|  | A34 | A34 |  | 150–200 | 20 |  | Yes | Yes |
|  |  |  | A34 | 80–140 |  | 0 | Yes | Yes |
|  | B5 | B5 |  | 150–200 | -20 |  | Yes | Yes |
|  |  |  | B5 | 80–130 |  | -25 | Yes | Yes |
|  |  |  | B6 | 80–140 |  | -25 | Yes | Yes |
| 3 | A9 | A7 |  | 150–200 | 5 |  | Yes | Yes |
|  |  | A8 |  | 150–200 | 5 |  | Yes | Yes |
|  |  |  | A33 | 80–130 |  | -10 | Yes | Yes |
|  | A35 | A7 |  | 150–200 | -10 |  | Yes | Yes |
|  |  | A35 |  | 150–200 | -10 |  | Yes | Yes |
|  |  |  | A35 | 80–130 |  | -25 | Yes | Yes |
| 4 | A7 | A6 |  | 150–200 | 15 |  | Yes | Yes |
|  |  |  | A6 | 80–150 |  | 15 | Yes | Yes |
| 5 | A3 | A3 |  | 150–190 | -10 |  | Yes | Yes |
|  |  |  | A3 | 80–130 |  | -20 | Yes | Yes |
|  | A18 | A14 |  | 150–200 | 15 |  | Yes | Yes |
|  |  |  | A46 | 80–120 |  | 0 | Yes | Yes |
|  | B11 | A18 |  | 150–190 | -25 |  | Yes | Yes |
|  |  |  | A18 | 80–150 |  | -25 | Yes | Yes |
| 6 | A6 | B2 |  | 160–200 | 15 |  | **No** | Yes |
|  |  |  | A6 | 100–150 |  | -10 | Yes | Yes |
| 7 | B4 | B2 |  | 160-200 | -30 |  | Yes | Yes |
|  |  |  | A19 | 80–140 |  | -65 | Yes | Yes |
|  | B30 | B13 |  | 150–200 | -40 |  | Yes | Yes |
|  |  | B19 |  | 150–190 | -40 |  | Yes | Yes |
|  |  |  | B13 | 80–140 |  | -115 | Yes | Yes |
| 8 | A2 | A2 |  | 150–190 | 5 |  | Yes | Yes |
|  |  |  | A2 | 80–150 |  | 5 | Yes | Yes |
| 9 | A38 | A33 |  | 150–200 | -15 |  | Yes | Yes |
|  |  |  | A33 | 80–120 |  | -20 | Yes | Yes |
| 10 | A6 | A6 |  | 150–190 | 5 |  | Yes | Yes |
|  |  |  | A6 | 80–130 |  | -5 | Yes | Yes |
| 11 | A1 | A40 |  | 150–190 | -25 |  | Yes | Yes |
|  |  |  | A40 | 80–130 |  | -125 | Yes | Yes |
|  | A38 | A31 |  | 150–200 | 5 |  | Yes | Yes |
|  |  | A32 |  | 160–200 | 5 |  | Yes | Yes |
|  |  | A38 |  | 150–200 | 5 |  | Yes | Yes |
|  |  |  | A31 | 80–130 |  | 0 | Yes | Yes |
|  |  |  | A32 | 80–130 |  | 0 | Yes | Yes |
|  |  |  | A33 | 80–130 |  | 0 | Yes | Yes |
|  |  |  | A38 | 80–150 |  | 0 | Yes | Yes |
| 12 | A23 | A23 |  | 160–200 | **0** |  | Yes | Yes |
|  |  |  | A23 | 80–150 |  | **10** | Yes | Yes |

Table S2. Comparison of ripples onset channels and their latency between 80–150 Hz and 150–200 Hz
