# Supplementary material for: The Onset of Interictal Spike-Related Ripples Facilitates Detection of the Epileptogenic Zone
Source: Front Neurol. 2021 Nov 4;12:724417. doi: 10.3389/fneur.2021.724417 (PMC8599368; doi:10.3389/fneur.2021.724417)
Supplement: Supplementary file 3 [file Table_3.DOCX]

|  | Patient | Pathology | Resection area | Ictal onset channnel | Ripple onset channel | The rate of coincided distribution of resection area and ictal onset channels |
| --- | --- | --- | --- | --- | --- | --- |
| **The rate of coincided distribution of ripple onset channels and ictal onset channels: 100%** | | | | | | |
|  | 8 | inflammatory change | Rt T and  hippocampus | A1, A2, A3, A7, A8, A9, A10, | A2 | 100% |
|  | 10 | FCD  hippocampal sclerosis | Lt T and  hippocampus | A4, A6, A44 | A6 | 100% |
|  | 12 | FCD | Rt F, T | A23, A24, A33 | A23 | 100% |
| **The rate of coincided distribution of ripple onset channels and ictal onset channels: 50%** | | | | | | |
|  | 1 | cortical tuber | Rt P, T, O | A32, A33, A34, A37, A38 | A35, A39 | **60%** |
| **The rate of coincided distribution of ripple onset channels and ictal onset channels: 0%** | | | | | | |
|  | 2 | FCD | Lt P, T, O and  hippocampus | A1, A2, A25, A26 | A13, A34, B5, B6 | 100% |
|  | 3 | FCD | Lt P, T, O and  hippocampus | A37, A43, A44 | A33, A35 | 100% |
|  | 4 | FCD | Rt F | A1, A6, A7 | A6 | 100% |
|  | 5 | hippocampal sclerosis | Lt F, P, T and  hippocampus | A1, A6, A11, A16, B7, B13 | A3, A46, A18 | 100% |
|  | 6 | FCD  hippocampal sclerosis | Lt T and  hippocampus | A11, A12 | A6 | 100% |
|  | 7 | FCD | Rt F, bilateral T and  hippocampus | B7, B8 | B19, B13 | 100% |
|  | 9 | oligodendroglioma | Rt P, T, O | B47 | A33 | 100% |
|  | 11 | FCD  hippocampal sclerosis | Rt F, bilateral T and  hippocampus | A1, A2, A3, A4, A6, A7, A8, A9, A10 | A40, A31, A32, A33, A38 | 100% |

F, frontal; FCD, focal cortical dysplasia; Lt, left; O, occipital; P, parietal; Rt, right; T, temporal

Table S3. The rate of coincided distribution of resection area, the ripple onset channels and ictal onset channels
