# Supplementary figures and images for: The Onset of Interictal Spike-Related Ripples Facilitates Detection of the Epileptogenic Zone
Source: Front Neurol. 2021 Nov 4;12:724417. doi: 10.3389/fneur.2021.724417 (PMC8599368; doi:10.3389/fneur.2021.724417)

## Slide 1
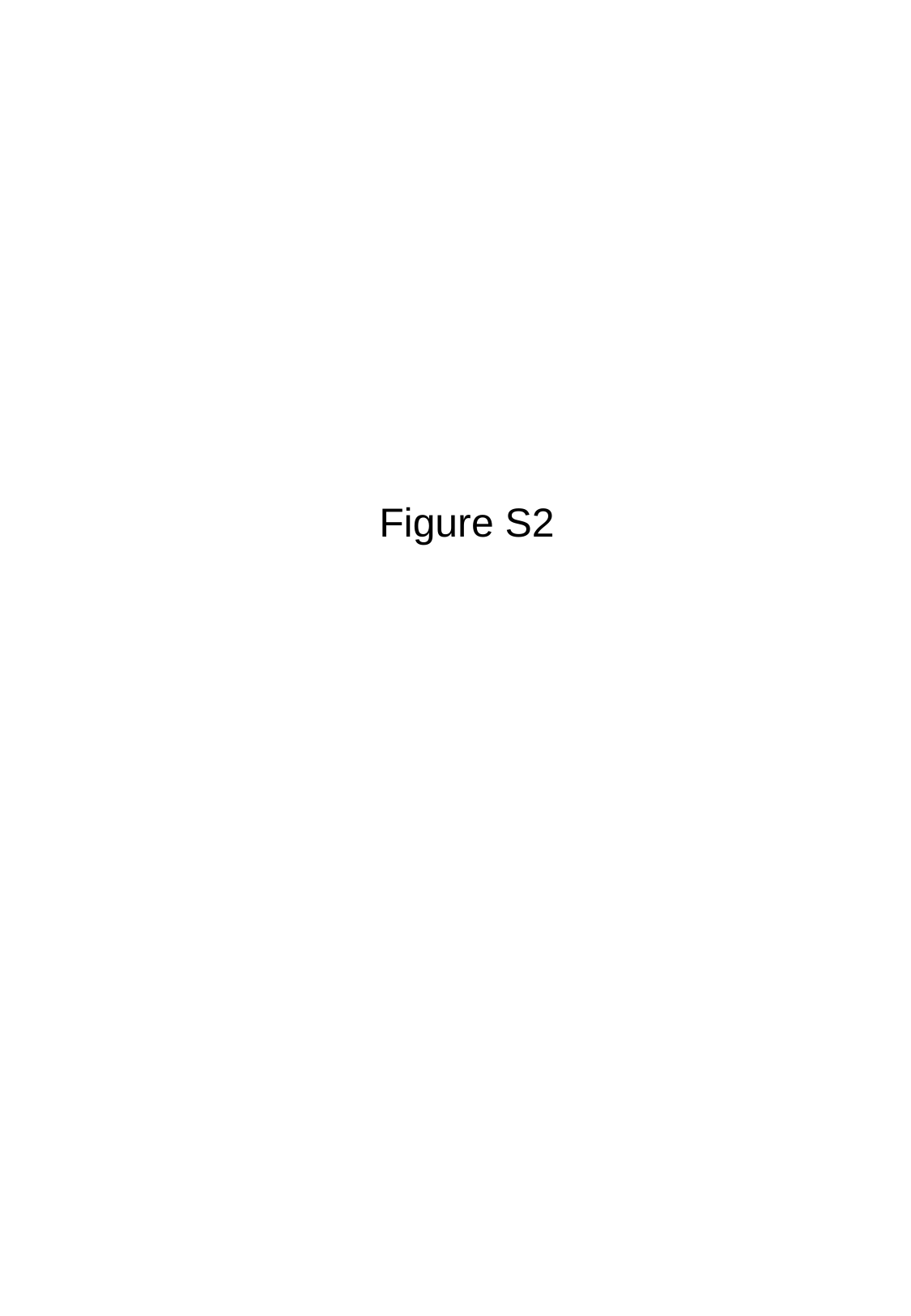

Figure S2

## Slide 2
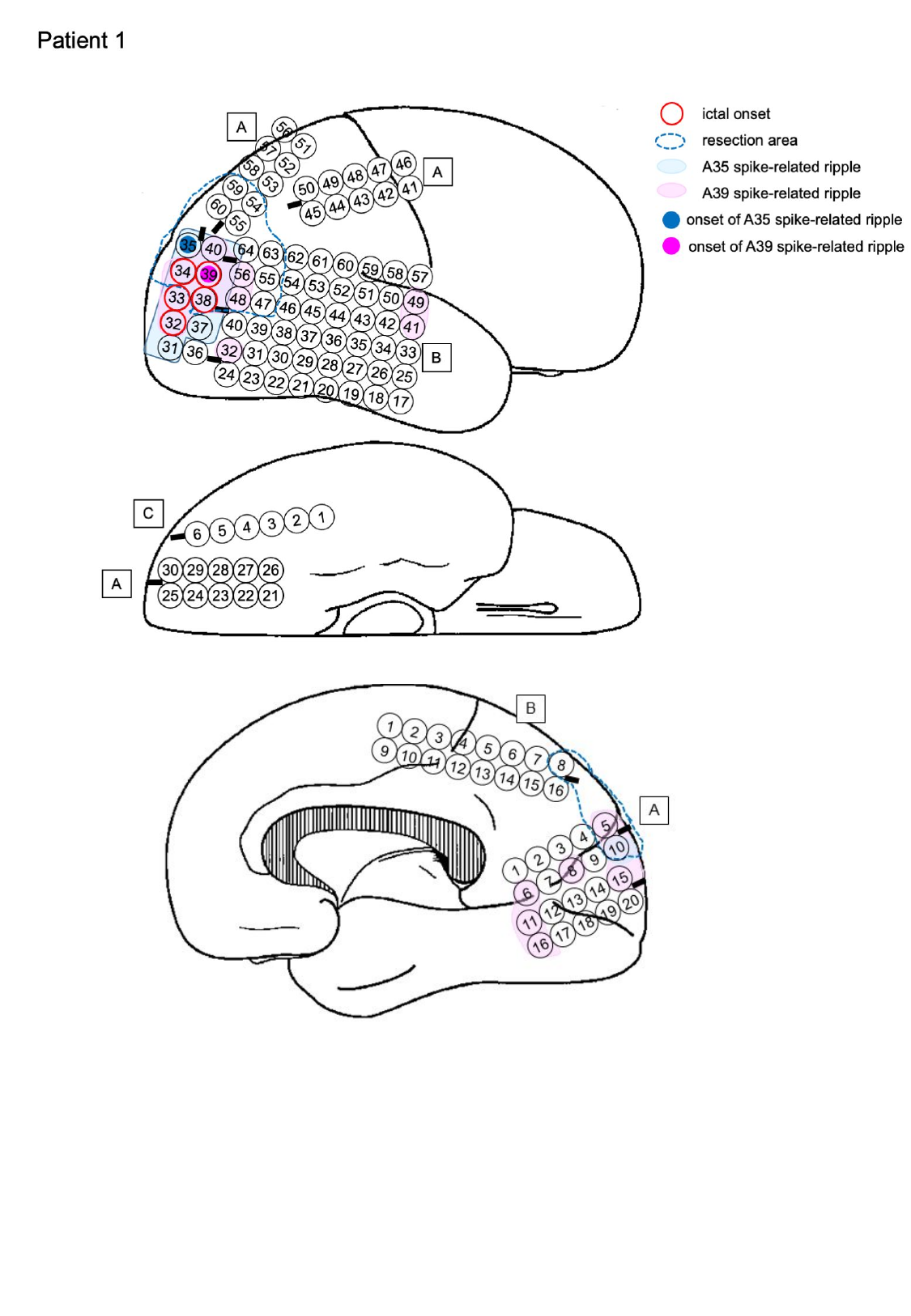

## Slide 3
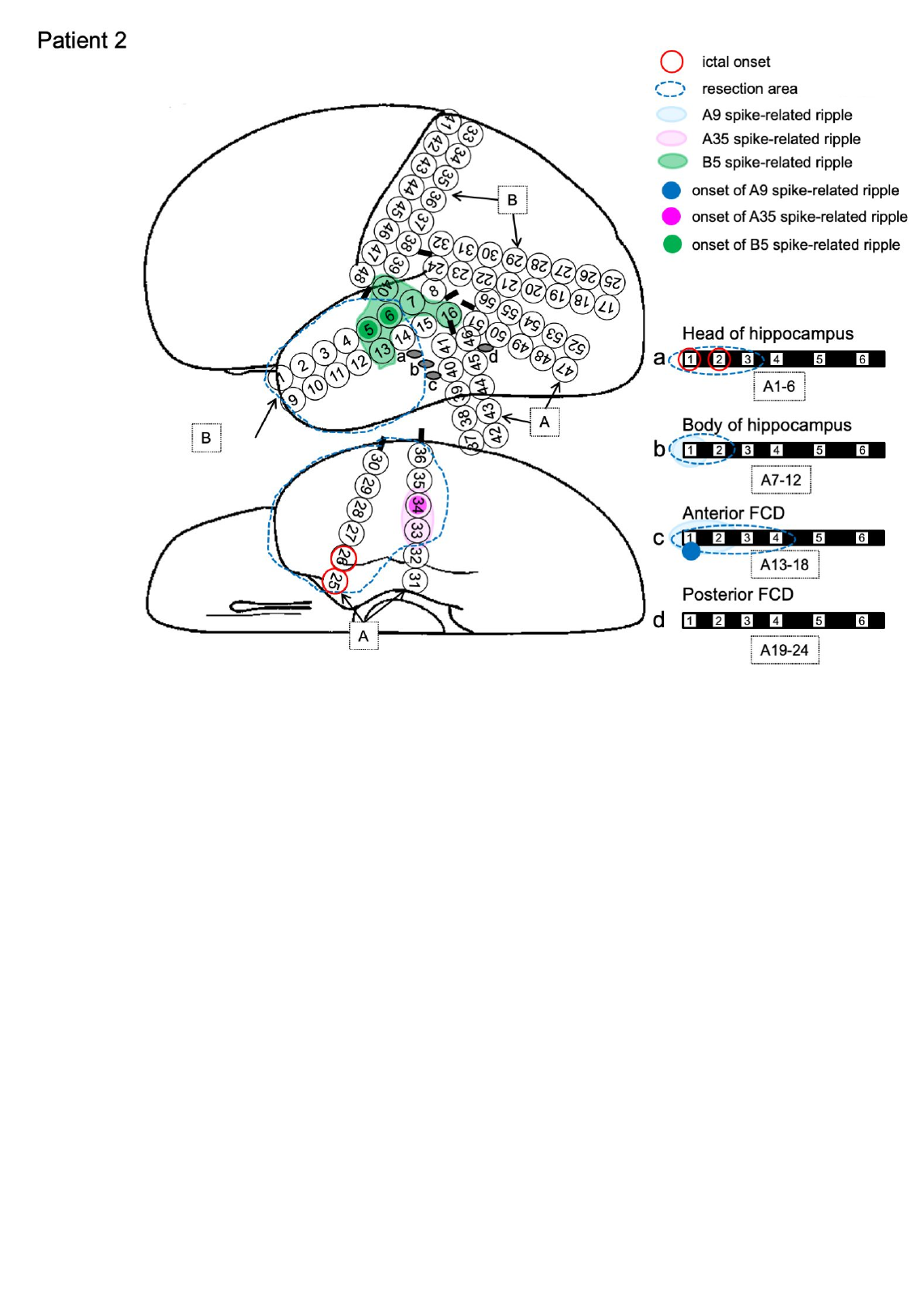

## Slide 4
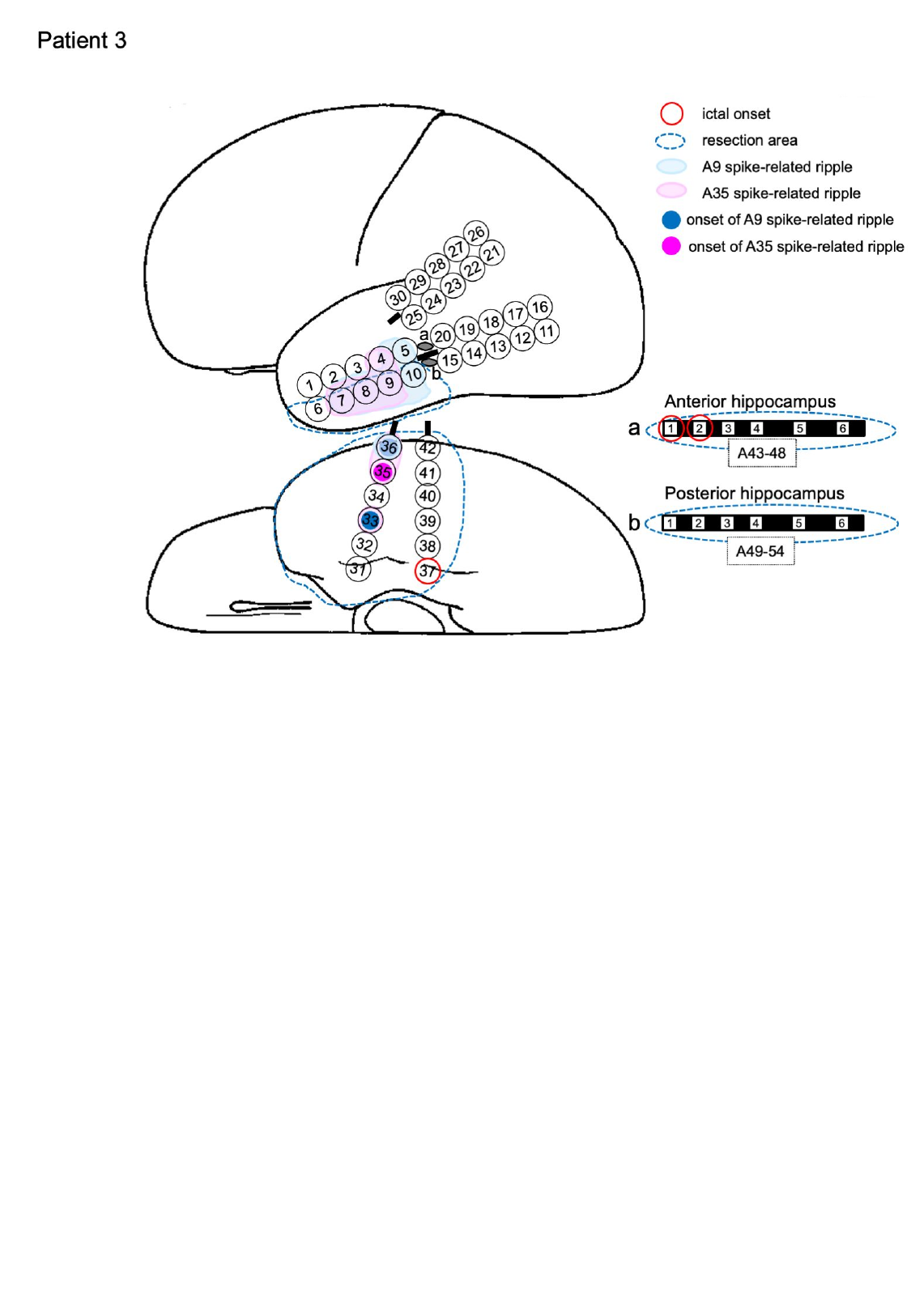

## Slide 5
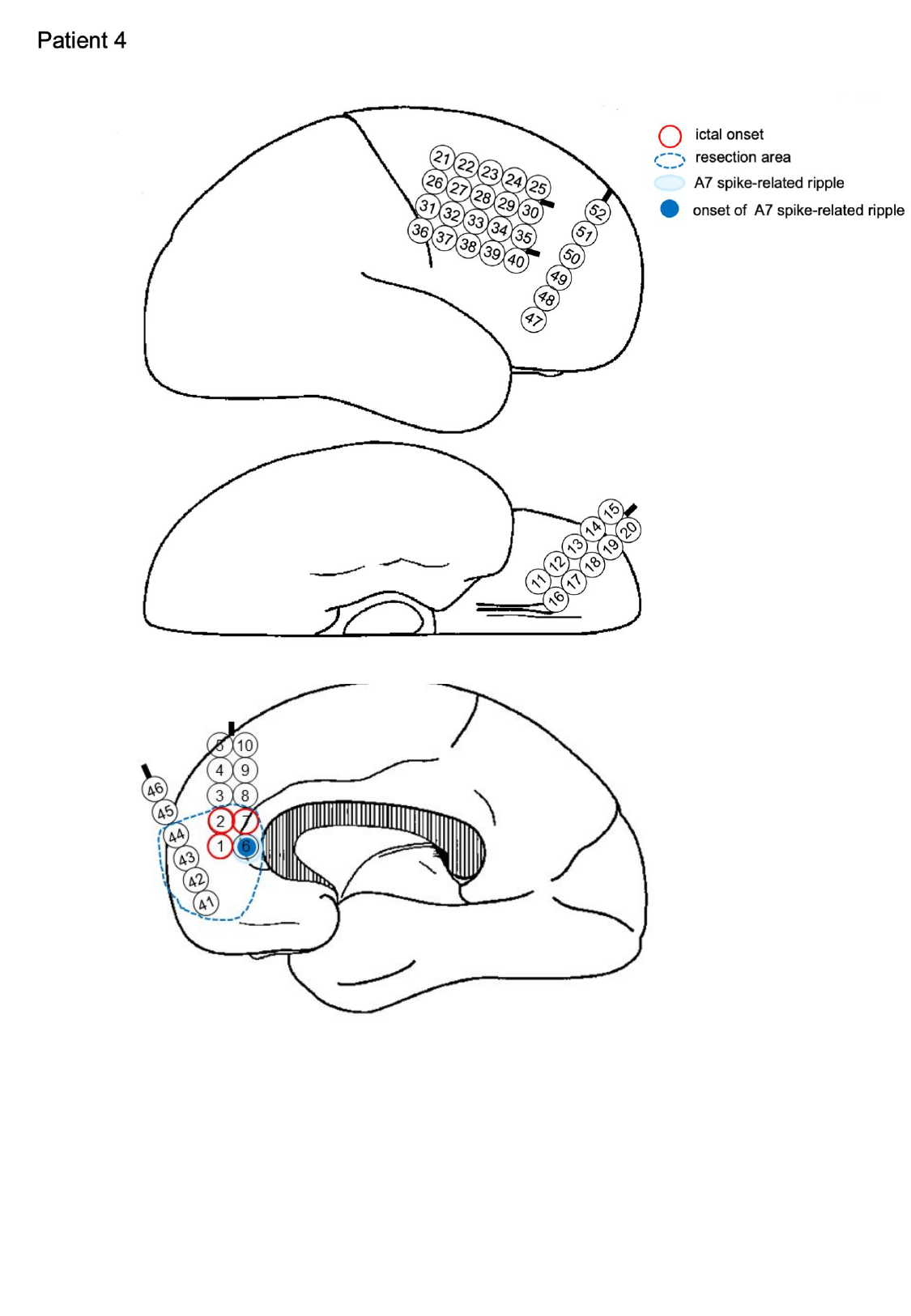

## Slide 6
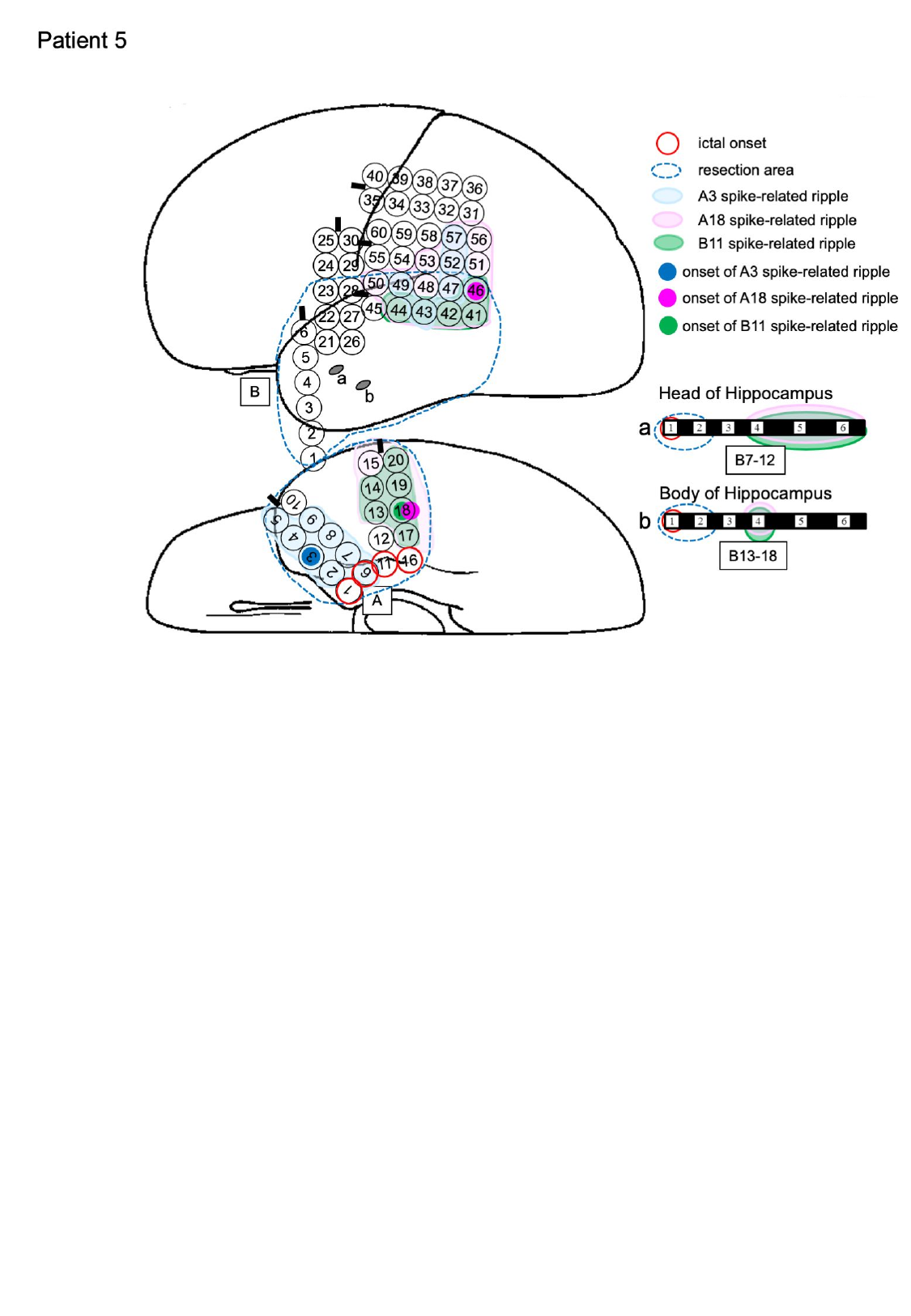

## Slide 7
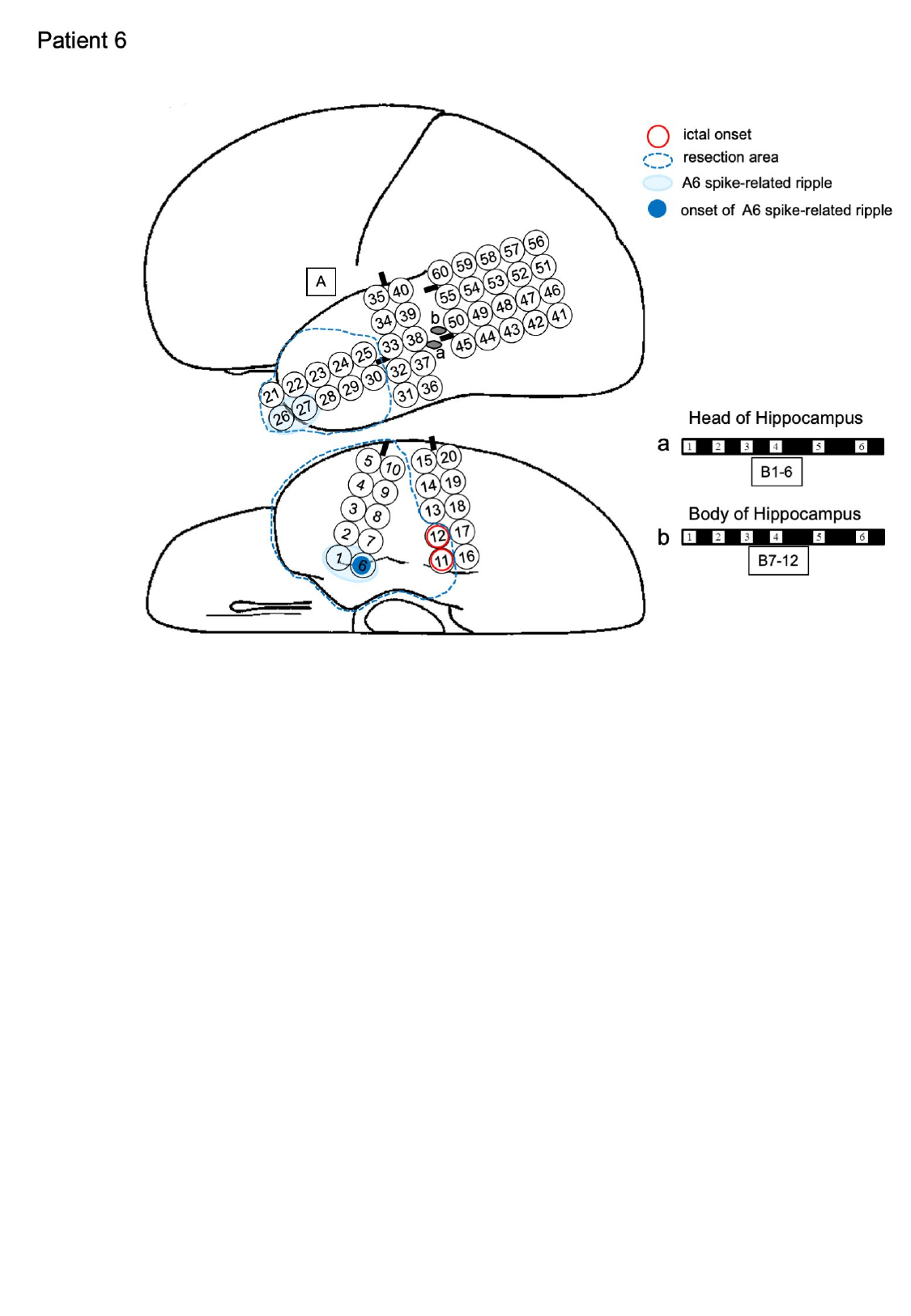

## Slide 8
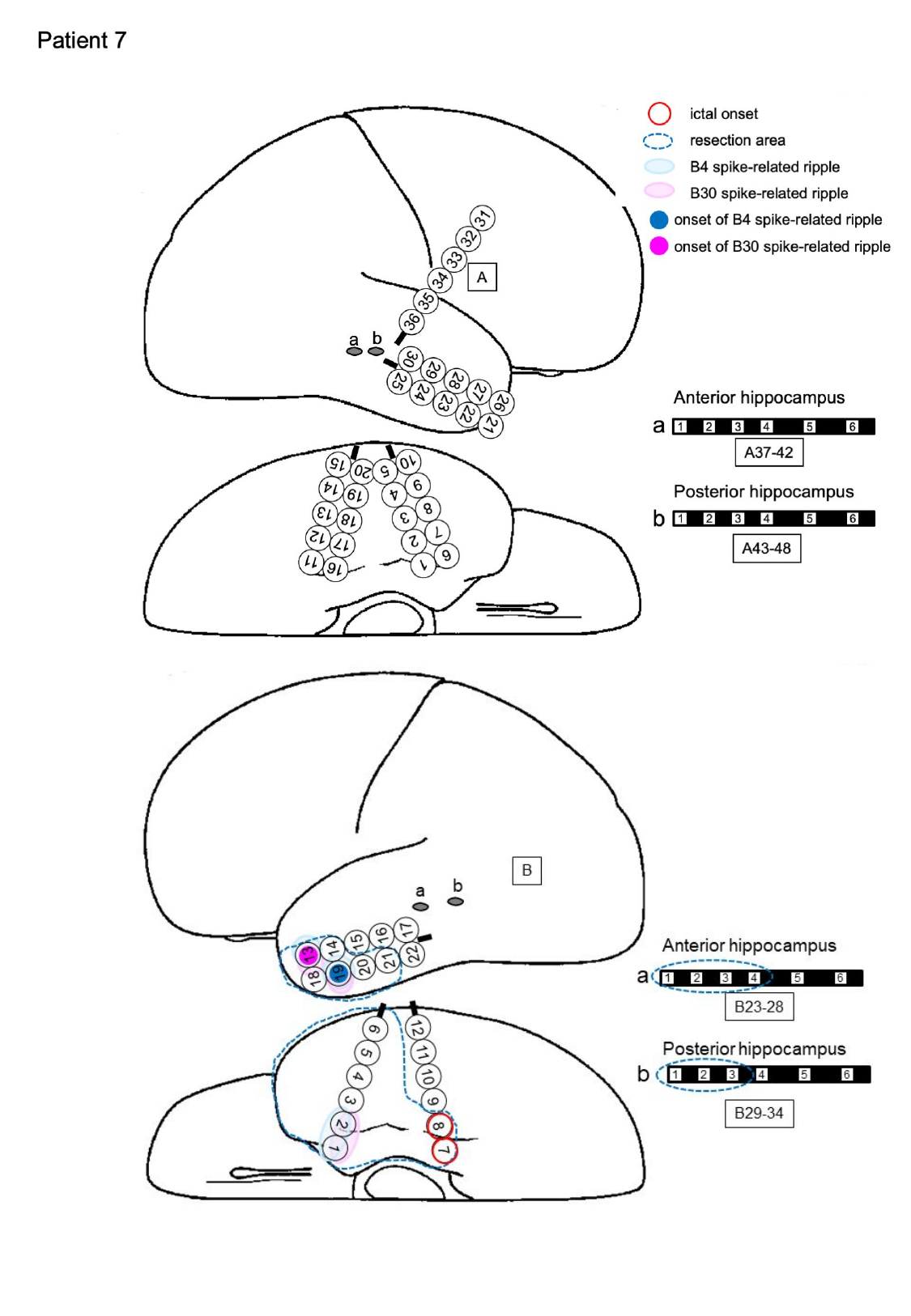

## Slide 9
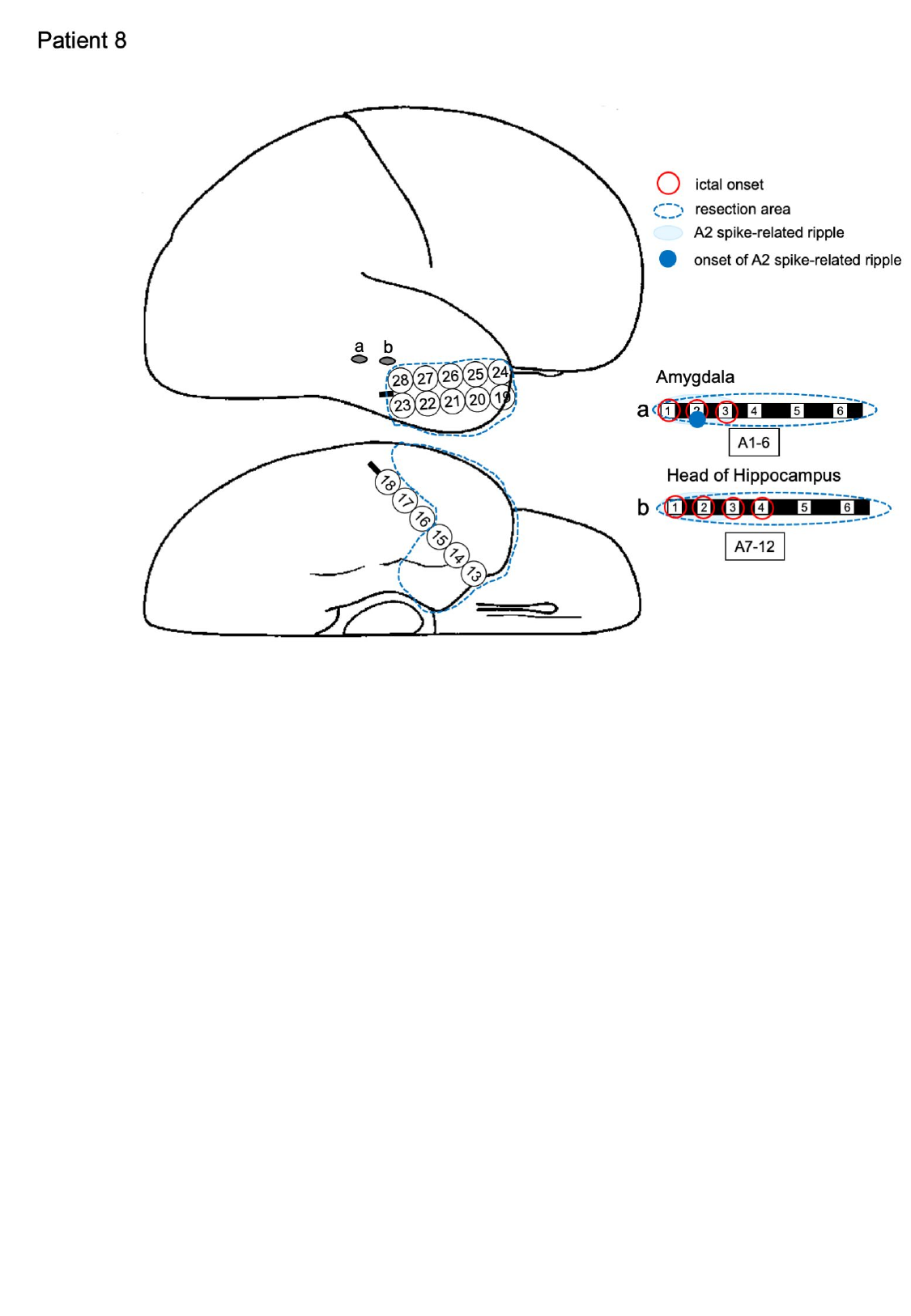

## Slide 10
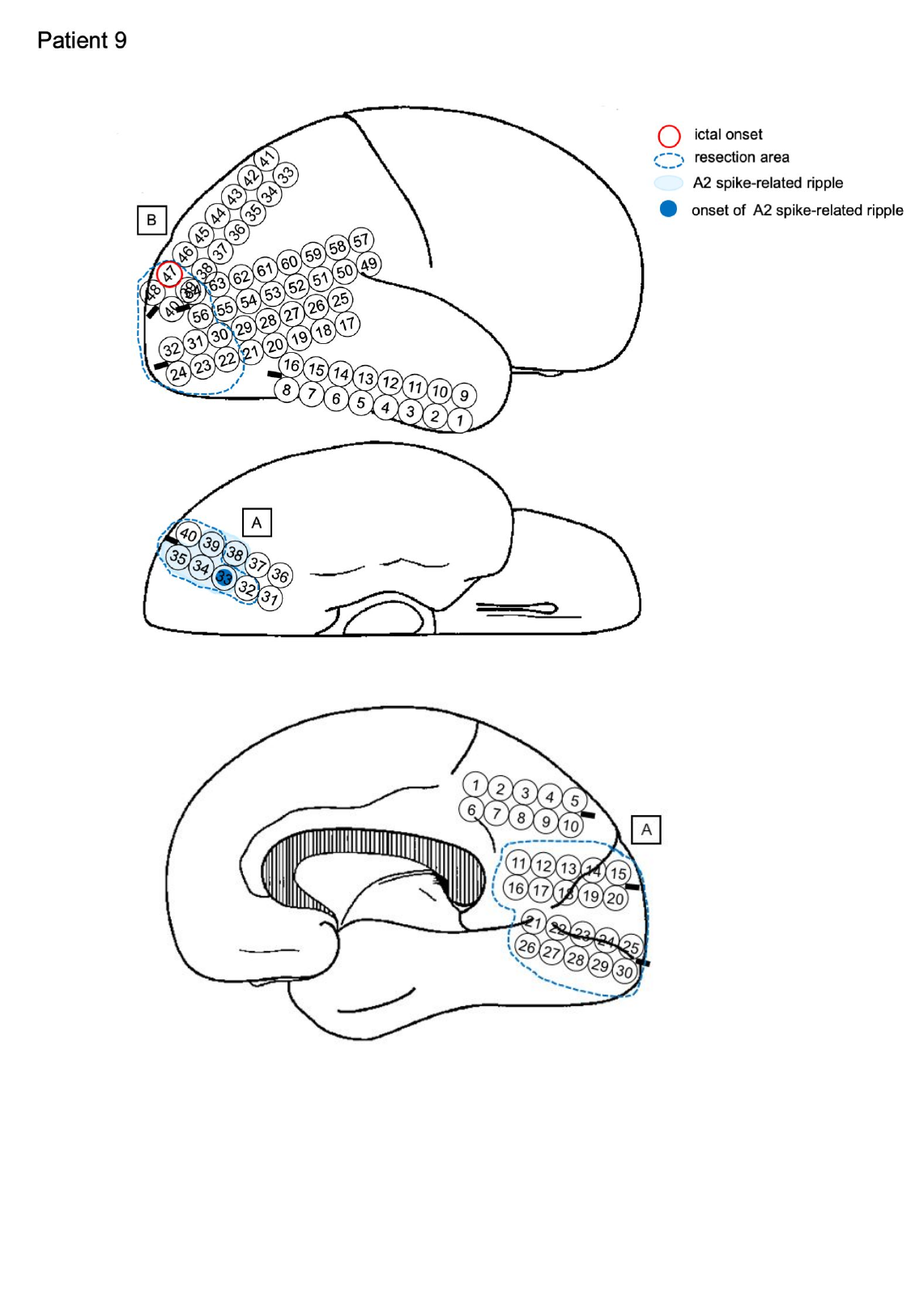

## Slide 11
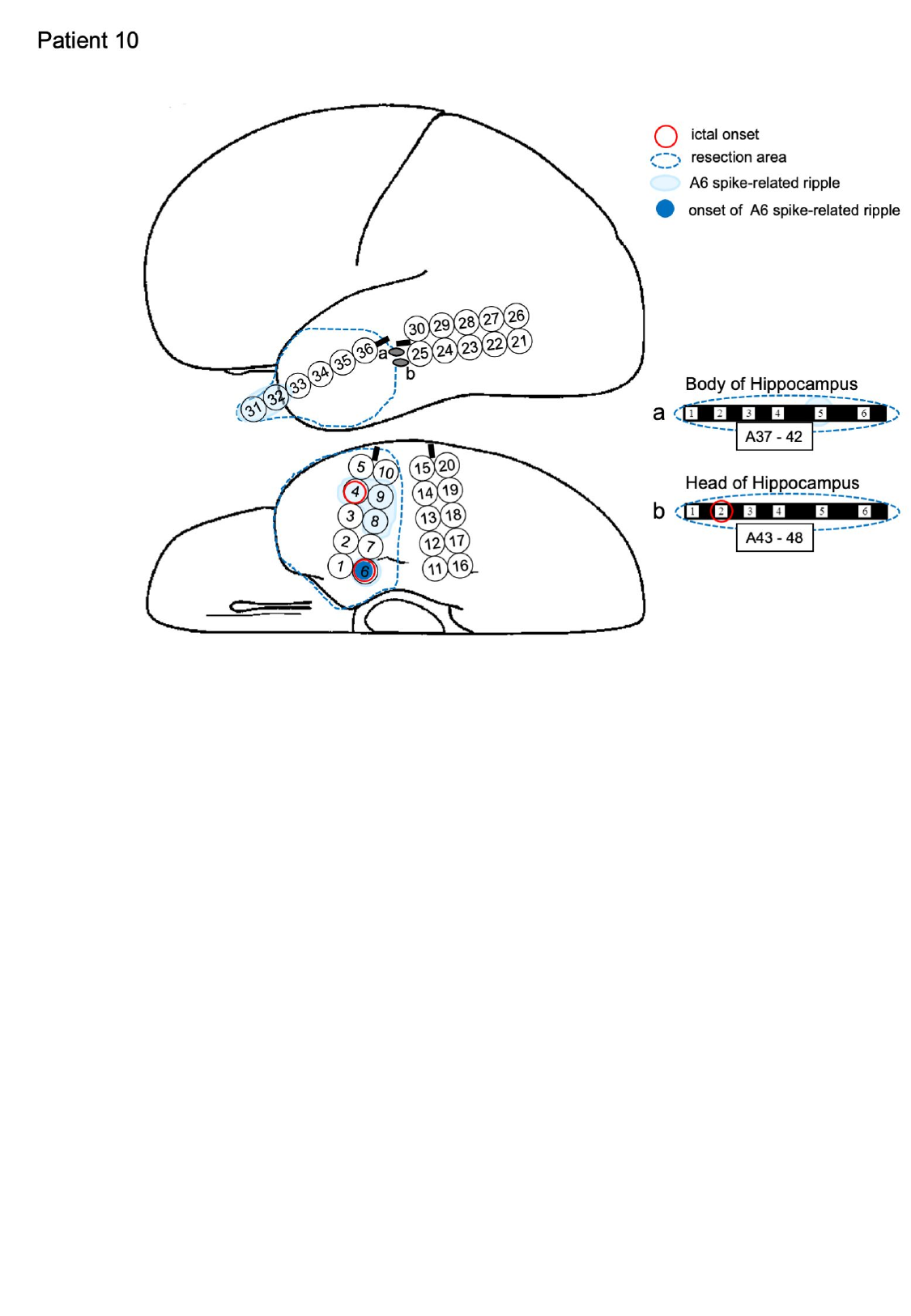

## Slide 12
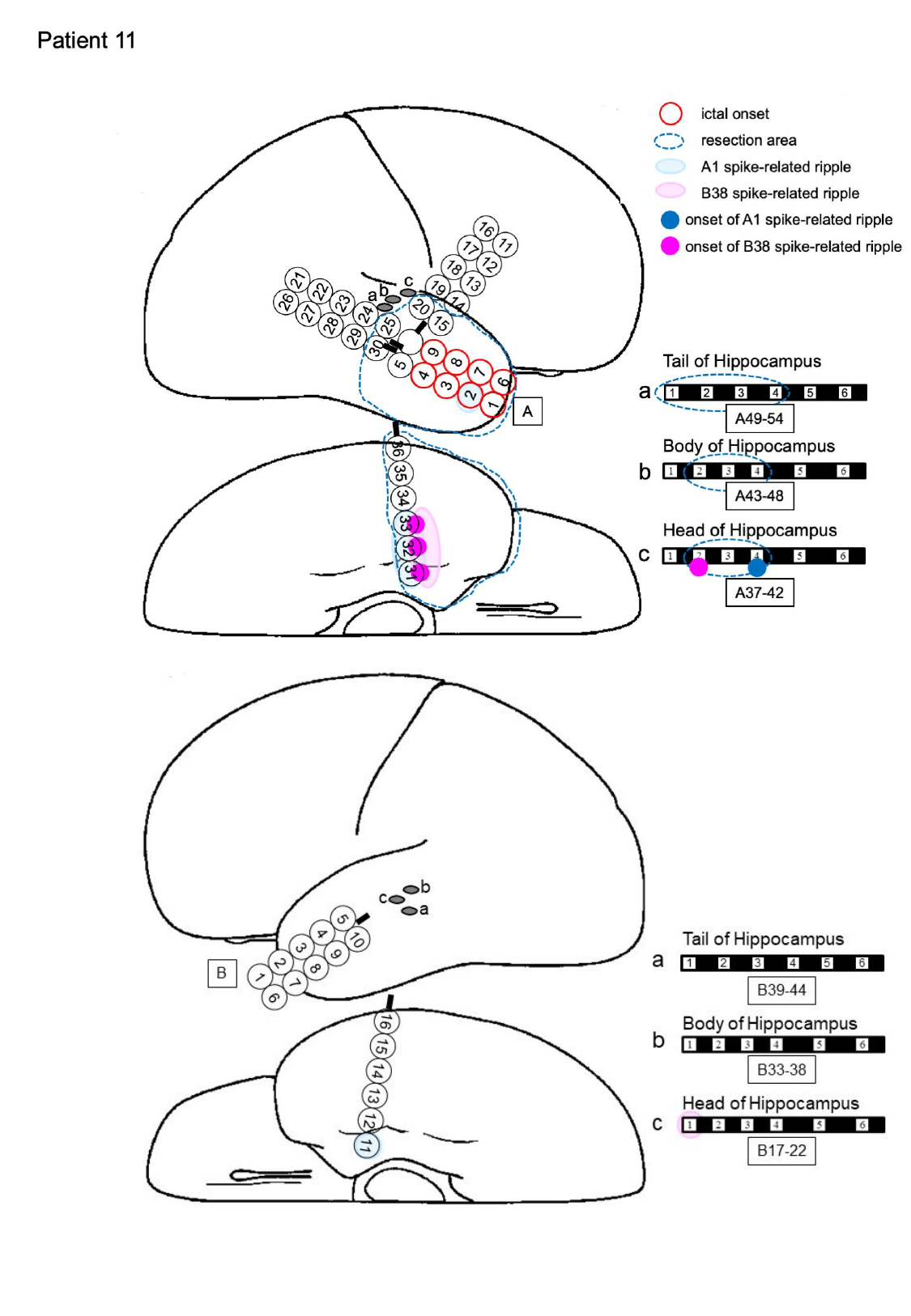

## Slide 13
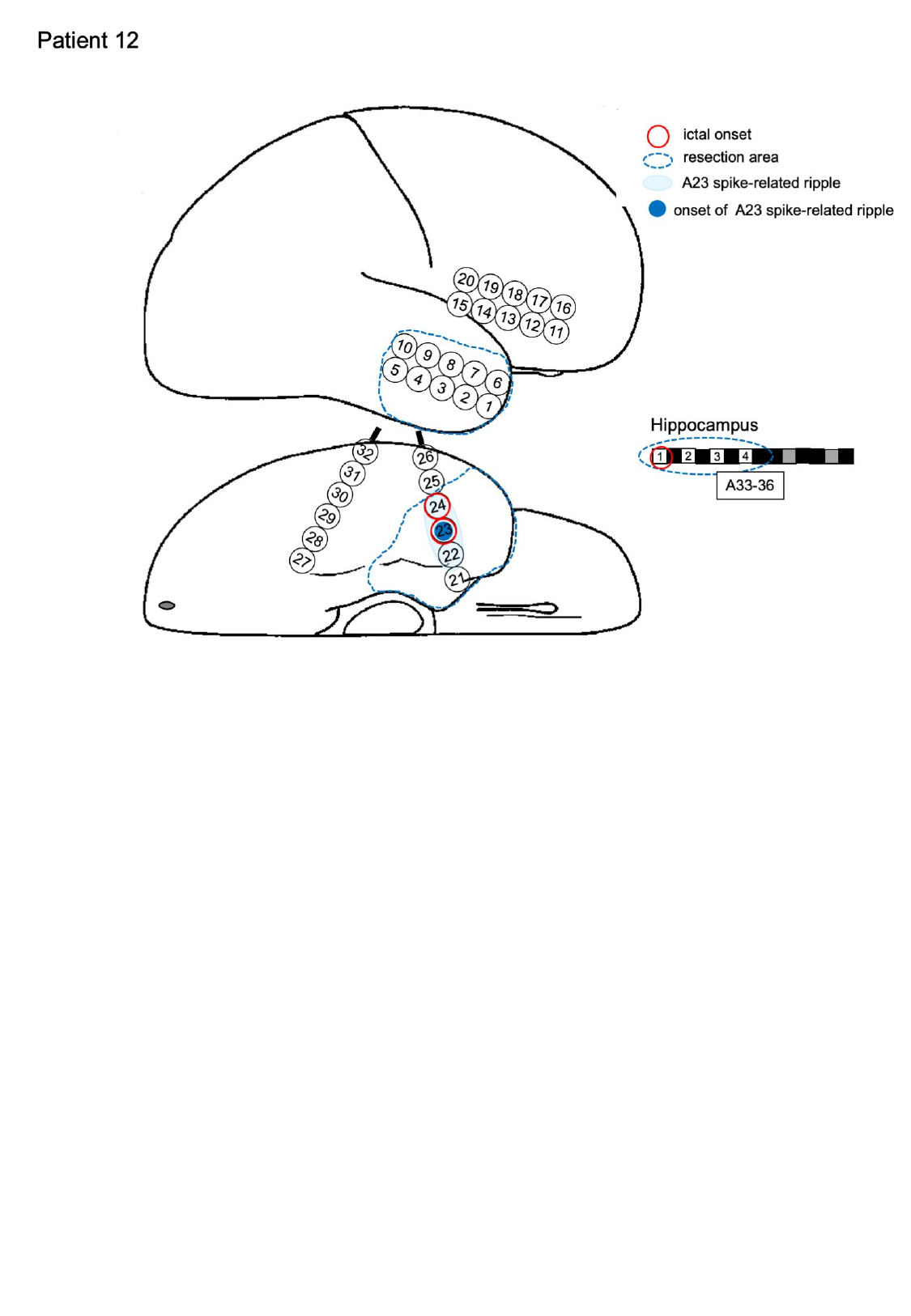

Supplement: Supplementary file 4 [file Presentation_1.PPT]

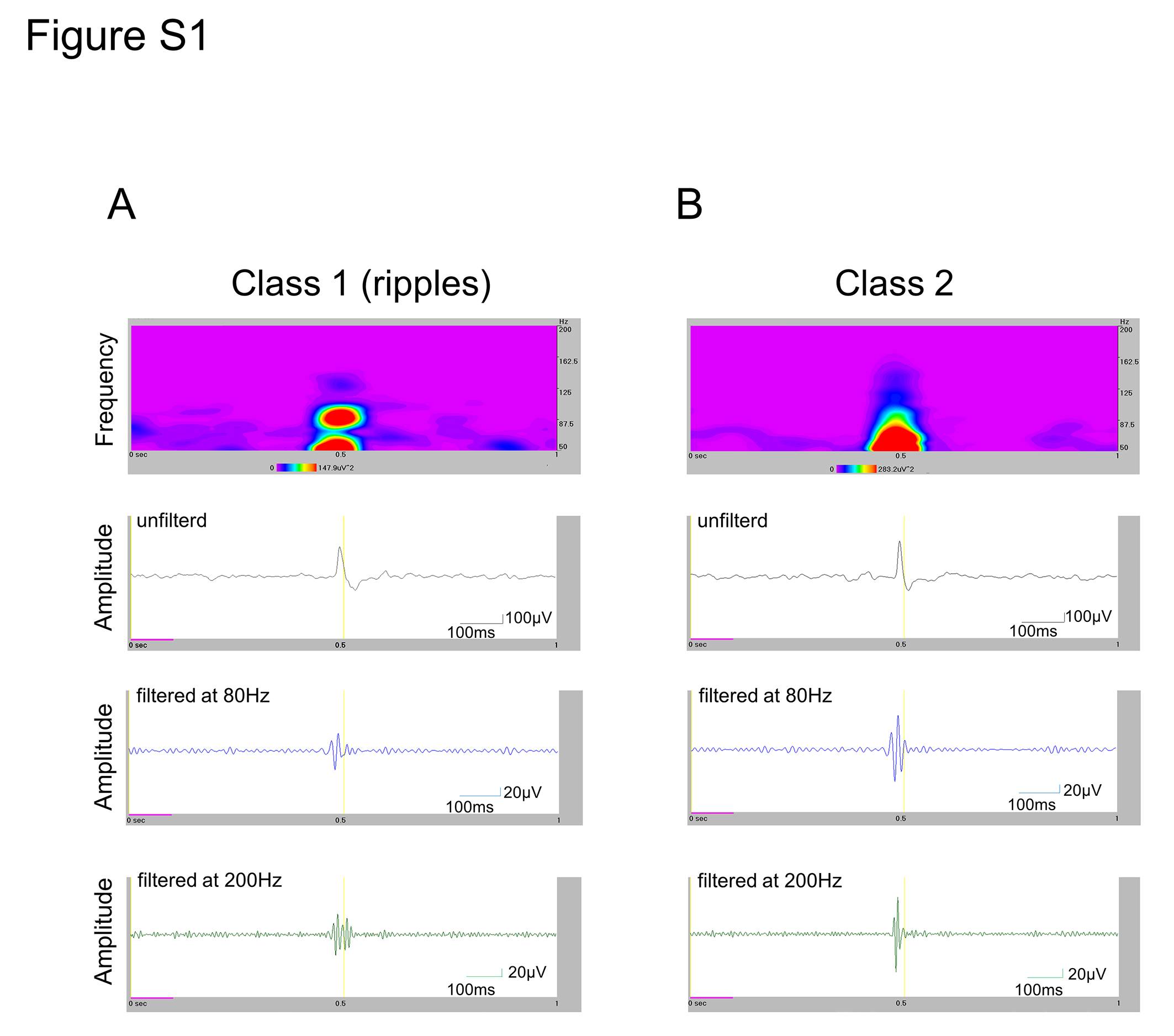

Supplement: Supplementary file 6 [file Image_1.TIF]
