# Supplementary material for: The Onset of Interictal Spike-Related Ripples Facilitates Detection of the Epileptogenic Zone
Source: Front Neurol. 2021 Nov 4;12:724417. doi: 10.3389/fneur.2021.724417 (PMC8599368; doi:10.3389/fneur.2021.724417)
Supplement: Supplementary file 5 [file Data_Sheet_1.DOCX]

**Figure S1. High-pass filtering for epileptic gamma activity**

Two types of spike-related gamma activities are shown. The top row shows the time-frequency analysis using the Wide Band EEG analysis Tool (Nihon Kohden’s EEG Suite, Neurofax, version 05-03), the second row shows the unfiltered interictal spikes, the third row shows the waveform when the high pass filter is set to 80 Hz, and the bottom row shows the waveform when the high pass filter is set to 200 Hz. (A) "Class 1" gamma activity is visible as a “blob” in the time-frequency plane (ripples). (B) “Class 2” gamma activity is considered no blob in the time-frequency plane and may be produced by spikes not related to gamma activity or artifacts.

**Figure S2. All analyzed findings superimposed with electrode placement schemas in all 12 patients**

The onset electrodes of spike-related ripples are shown as filled circles (blue, pink and green). Ictal onset electrodes are shown as red circles. Spike-related ripples are shown as light pink, light blue and light green areas. The area of the resection margin is drawn as a blue dotted line.
